# Nanoporous-based biomaterials in biomedical applications: from fundamentals and biosensing to drug delivery, wound healing, and tissue engineering

**DOI:** 10.1039/d5na00451a

**Published:** 2026-06-11

**Authors:** Zahra Saadatidizaji, Iman Zare, Mingzhen Zhang, Shahrzad Zirak Hassan Kiadeh, Mojdeh Mirshafiei, Amin Farahani, Zahra Sadat, Hooman Aghamirza Moghim Aliabadi, Mohammad Mahdavi, Reza Eivazzadeh-Keihan, Amir Kashtiaray, Parisa Rezvani, Aisan Yahyapoor, Amirali Vaziri, Hooriehsadat Amini, Meng Yu

**Affiliations:** a Faculty of Chemistry, University of Tabriz 51666-16471 Tabriz Iran; b Research and Development Department, Sina Medical Biochemistry Technologies Co., Ltd. Shiraz 7178795844 Iran; c School of Basic Medical Sciences, Xi'an Jiaotong University Xi'an Shaanxi 710061 China; d Key Laboratory of Environment and Genes Related to Diseases, Xi'an Jiaotong University Xi'an Shaanxi 710061 China; e New Technologies Research Center (NTRC), Amirkabir University of Technology (Tehran Polytechnic) Tehran Iran; f Department of Biotechnology, School of Chemical Engineering, College of Engineering, University of Tehran Tehran Iran; g Cellular and Molecular Endocrine Research Center, Research Institute for Endocrine Sciences, Shahid Beheshti University of Medical Sciences Tehran Iran; h Endocrinology and Metabolism Research Center, Endocrinology and Metabolism Clinical Sciences Institute, Tehran University of Medical Sciences Tehran Iran reza_eivazzadeh@chem.iust.ac.ir amir.inventor@gmail.com; i Advanced Chemical Studies Lab, Department of Chemistry, K. N. Toosi University of Technology Tehran Iran; j Faculty of Paramedical Sciences, Tehran Medical Sciences, Islamic Azad University Tehran Iran; k Faculty of Veterinary Medicine, Islamic Azad University Alborz Iran; l Department of Medicinal Chemistry, Faculty of Pharmacy, Tehran Medical Sciences Branch, Islamic Azad University Tehran Iran; m NMPA Key Laboratory for Research and Evaluation of Drug Metabolism & Guangdong Provincial Key Laboratory of New Drug Screening & Guangdong-Hongkong-Macao Joint Laboratory for New Drug Screening, School of Pharmaceutical Sciences, Southern Medical University Guangzhou 510515 China yumeng999@smu.edu.cn

## Abstract

Nanoporous materials (NPMs) have emerged as powerful tools in the fields of medicine, revolutionizing diagnostics, treatment, and patient care. Their unique properties address critical challenges in healthcare. Nanoporous matrices offer abundant active sites for molecular adsorption, catalysis, and interactions with biological entities. Efficient loading of therapeutic agents and biomolecules occurs due to their expansive surface area. Precise control over pore dimensions allows selective sieving, crucial for drug delivery and biosensors. Their surface chemistry and mechanical properties play pivotal roles in ensuring biocompatibility for applications like tissue scaffolds and implantable devices. In this context, the properties of NPMs, including physicochemical properties (such as crystallinity and mechanical properties), drug release and adsorption, and biological characteristics, have been comprehensively discussed. Furthermore, we also explore the multifaceted applications of NPMs, focusing on biosensing, drug delivery, wound healing, and tissue engineering.

## Abstract 2

Nanoporous materials (NPMs) have emerged as powerful tools in the fields of medicine, revolutionizing diagnostics, treatment, and patient care.

## Graphical abstract

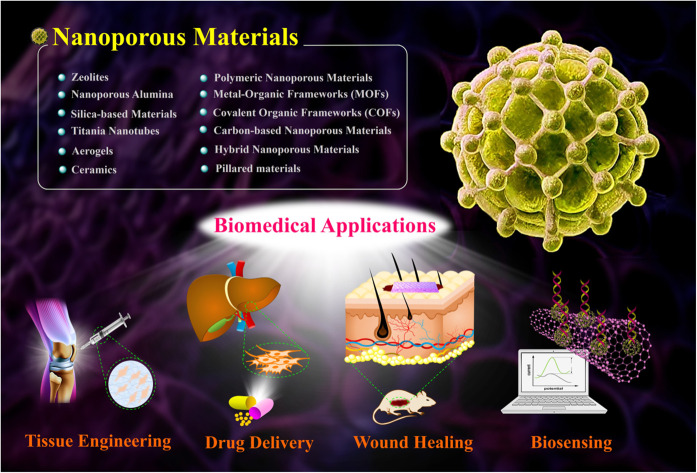

## 1. Introduction

NPMs have emerged as versatile tools with immense potential in various biomedical applications.^1^ They have pores with diameters of typically 100 nanometers or smaller. These pores are categorized based on their size into three categories: micropores (<2 nm), mesopores (2–50 nm), and macropores (>50 nm).^2^ NPMs exhibit a diverse range of tunable properties; for instance, at the heart of NPMs lies their expansive surface area. Unlike bulk materials, which have limited interaction sites, nanoporous matrices provide an abundance of active sites for molecular adsorption, catalysis, and biological applications.^3–5^ This high surface-to-volume ratio facilitates the efficient loading of therapeutic agents, biomolecules, and signaling cues. Whether it is drug molecules seeking binding sites or enzymes catalyzing biochemical reactions, NPMs offer an ideal platform for innovation.^6,7^ The ability to tailor pore sizes within NPMs is a game-changer.^8^ Researchers can engineer these materials to accommodate specific molecules, cells, or even viruses. By precisely controlling pore dimensions, they can achieve selective sieving, favoring the passage of desired species while excluding others. This tunability is crucial for drug delivery systems (DDSs), where controlled release kinetics and site-specific targeting are paramount.^9^ Moreover, in biosensors, nanoporous membranes serve multiple critical functions. They act as molecular sieves, selectively allowing analytes of interest to pass through, which improves the selectivity of the sensor. Additionally, these membranes function as carriers for the immobilization of enzymes, proteins, or signal molecules, which are essential for the biosensing process. Additionally, the concentration of analytes in nanoporous membranes can improve sensitivity. These multifunctional roles of nanoporous membranes contribute significantly to the improved performance of biosensors.^10–13^ Biocompatibility is non-negotiable in biomedical applications. NPMs, when designed appropriately, exhibit minimal cytotoxicity and favorable interactions with biological tissues. Their surface chemistry, charge distribution, and mechanical properties play pivotal roles in determining biocompatibility. From implantable devices to tissue scaffolds, NPMs seamlessly integrate with living systems. For instance, mesoporous silica nanoparticles (NPs), when functionalized with biocompatible coatings, can navigate biological barriers and deliver payloads precisely to target sites.^14,15^

The distinctive characteristics of NPMs contribute significantly to their utility in various biomedical applications. Nanoporous carriers enable sustained drug release, personalized dosing, and reduced side effects.^16,17^ By adjusting pore size and surface properties, they enable precise drug delivery to specific tissues or cells. This targeted approach minimizes off-target effects and improves therapeutic efficacy.^18^ NPMs are used in biosensors for detecting biomolecules, pathogens, and disease markers. Their rapid response times and exquisite selectivity redefine diagnostics. Their large surface area allows efficient binding of target molecules, leading to sensitive and rapid detection.^19^ NPMs serve as supportive scaffolds for tissue regeneration and growth. Whether it is bone, cartilage, or skin, nanoporous matrices provide cues for cellular growth and differentiation. These structures promote cell adhesion, proliferation, and differentiation, contributing to tissue repair and regeneration. Researchers have developed intelligent nanoporous-based systems for tissue engineering, both in *in vitro* and *in vivo* environments. Nanoporous dressings accelerate wound closure, modulate inflammation, and promote tissue regeneration. Their multifunctionality addresses the complex dynamics of wound repair.^20–22^ In this context, the properties of NPMs, including crystallinity, mechanical and physicochemical properties, drug release and adsorption, and biological characteristics, are comprehensively discussed. Furthermore, we will delve into each application to unravel the promise and challenges of NPMs, envisioning a future where these tiny structures wield outsized influence in improving human health.

## 2. Characterization of nanoporous materials

Characterization of NPMs is essential for understanding their properties and optimizing their applications in the biomedical field. Key characterization techniques include X-ray diffraction (XRD) for crystallinity, Brunauer–Emmett–Teller (BET) analysis for specific surface area and porosity (including pore size distribution), mechanical testing for strength and elasticity, and various spectroscopic methods (*e.g.*, Fourier-transform infrared spectroscopy (FTIR), Raman spectroscopy, and X-ray photoelectron spectroscopy (XPS)) for chemical composition and biological interactions.

### 2.1. Physicochemical properties

The performance and applications of NPMs in catalysis, drug delivery, and environmental remediation are greatly affected by their physicochemical properties. These characteristics, encompassing physical, chemical, and structural aspects, dictate how these materials interact with their surroundings. Below is a summary of the key physicochemical properties of NPMs.

#### 2.1.1. Crystallinity

Crystallinity is defined as the presence of a structured, repetitive atomic arrangement in a substance. The degree of crystallinity refers to the extent to which a material exhibits this ordered structure.^23^ Materials can be categorized as crystalline, semi-crystalline, or amorphous depending on their structural arrangement. XRD offers insights into the crystalline phases, degree of crystallinity, and lattice parameters. Differential scanning calorimetry (DSC) is utilized for determining thermal transitions like melting points to indicate levels of crystallinity.^24^

The degree of crystallinity in nanoporous substances is an important feature that greatly impacts their properties in terms of biology, physics, chemistry, and mechanics.^25–28^ Below are a few important factors to consider regarding crystallinity in NPMs:

Various methods of synthesis (such as sol–gel, hydrothermal, *etc.*) result in different levels of crystallinity.^29,30^ For instance, controlled heating during synthesis can promote crystallization.^31^ In template-synthesized NPMs, the final crystallinity can be affected by the removal process. Residual templates can obstruct the arrangement of the crystal structure. Processes such as annealing have the capability to improve crystallinity by enabling atoms to reorganize into a more structured form.^32,33^ On the other hand, some post-synthesis treatments such as ion irradiation or high-energy mechanical milling could result in amorphization.^34,35^

Crystalline NPMs can offer predictable drug release kinetics by maintaining consistent pore structures, promoting controlled release profiles.^36^ The sensitivity and selectivity of biosensors can be impacted by the level of crystallinity, as the order of the material can impact its interactions with analytes. The crystallinity of nanopores also provides the capability to sense biomolecules.^37^ Crystalline materials can display varying optical characteristics when compared to amorphous materials, affecting photonic applications.^38^ Crystalline structures usually provide superior strength and longevity, crucial for applications that need structural integrity.

The level of crystallinity in NPMs is crucial in determining how well they perform and if they are suitable for certain uses. Adjusting the level and type of crystallinity during both production and post-treatment procedures can improve the characteristics of materials, making them suitable for a wide range of applications, including catalysis and biomedical engineering. The comprehension and regulation of crystallinity will remain a vital focus of study and advancement in the domain of materials science.

#### 2.1.2. Mechanical properties

The performance of NPMs in biomedicine and catalysis depends significantly on their mechanical properties.^20,39^ The porosity, pore structure, and overall composition of the material have a significant impact on these properties. Below are important mechanical characteristics to take into account:

NPMs frequently have lower compressive strength as a result of voids and decreased density. Nevertheless, their design can enable specific uses where lightweight materials are beneficial. While NPMs may have decreased tensile strength compared to solid materials, their specific structure can still offer sufficient strength for certain purposes, particularly when engineered with optimal pore morphology.

The existence of pores in NPMs can lead to a decrease in the elastic modulus by acting as points of stress concentration.^40^ The modulus may change based on the pore size and distribution, along with the material's total density.^41,42^ This characteristic could vary depending on different pore sizes and shapes, altering the way the material responds to stress. It is typically reduced in NPMs due to their irregular structure and reduced connectivity, which can lead to brittle fracture under strain. NPMs are often more susceptible to brittle fracture than non-porous materials, especially if they are predominantly amorphous.^43^

Mechanical characteristics may change depending on the temperature. Depending on their composition and structure, certain NPMs exhibit better strength retention at high temperatures compared to others. The chemical environment, particularly solvents or biological fluids, can affect the structural stability of NPMs. Surface characteristics are able to impact the mechanical properties as well, playing a role in adhesion and bonding in composite applications. Adding coatings or making surface modifications can improve mechanical characteristics, such as wear resistance and toughness.

The performance of NPMs in different applications depends greatly on their mechanical properties, like strength, elasticity, ductility, fatigue resistance, and impact toughness. Comprehending and enhancing these characteristics *via* design, regulation of pore arrangement, and material choice is crucial for creating efficient NPMs customized for particular requirements, whether in biomedical devices, catalysts, or lightweight structural components.

#### 2.1.3. Porosity

Porosity is the proportion of void spaces (pores) within a material. It significantly affects the material's surface area, storage capacity, and reactivity. Varieties of porosity include:

• Microporosity refers to pores that have a diameter of less than 2 nm, which are commonly seen in zeolites and activated carbons.

• Mesoporosity refers to pores with sizes ranging from 2 nm to 50 nm, often found in silica and metal–organic frameworks (MOFs).

• Macroporosity refers to pores that are greater than 50 nm in size, which are important in certain scaffolds used in biomedical applications.

#### 2.1.4. Surface area

The total surface area per unit mass of the material is often measured using techniques like Brunauer–Emmett–Teller (BET) analysis.^44^ Increased area on the surface improves the ability to absorb and react.^45^ A larger surface area allows for greater interaction with guest molecules, influencing drug loading, catalysis, and gas adsorption applications.^5,18,46^

#### 2.1.5. Chemical composition

NPMs can consist of a variety of substances, such as inorganic materials like silica and alumina, organic materials like polymers, or a combination of both. The stability, reactivity, and potential for functionalization of a material are determined by its chemical composition.

Having certain functional groups on the surface can improve chemical reactivity, enabling selective adsorption and precise interactions with biomolecules or drugs. Surface modifications can customize chemical reactivity, allowing for increased interactions with certain molecules or surroundings.

#### 2.1.6. Chemical stability

It is crucial for the long-term use of NPMs that they are able to withstand degradation or dissolution in harsh environments like acidic or basic conditions. The performance of NPMs in different applications is substantially affected by their physicochemical attributes like porosity, surface area, pore size, chemical composition, and mechanical and thermal properties.^47,48^ Researchers can create specialized materials for various applications, such as catalysis and biomedical engineering, by adjusting their properties using synthesis and modification methods. Comprehension of these characteristics is crucial for enhancing NPMs for their specific uses.

#### 2.1.7. Thermal and electrical properties

The capacity of NPMs to withstand high temperatures without degradation is necessary for their use in catalysis and environmental remediation.^49^ Understanding thermal conductivity is crucial when evaluating materials for thermal insulation or devices that depend on precise temperature management. Certain NPMs, particularly ones containing conductive elements, can exhibit notable electrical conductivity, which is advantageous for biosensing.^19^ The utilization of NPMs in electronic devices can be affected by the dielectric constant and breakdown voltage.

### 2.2. Drug release and adsorption

The release of drugs and their adsorption are crucial roles of NPMs, especially in DDSs that require controlled release and successful adsorption of therapeutic substances.^50–52^ NPMs are appropriate for these uses due to their distinct characteristics, including high surface area, customizable pore size, and durable structure. Here is a summary of the processes and elements that impact the release and adsorption of drugs in NPMs.

#### 2.2.1. Drug release mechanisms

This is the predominant mechanism by which drug molecules diffuse through the pores of NPMs. Factors such as pore size, drug molecular size, and system temperature impact the rate of release.^53^ Swelling may occur in systems with NPMs that are hydrophilic or contain hydrophilic polymers when they come into contact with bodily fluids, causing drug release as the matrix expands.^54–56^ NPMs can release encapsulated drugs triggered by exogenous stimuli like pH, temperature, or light.^57,58^

Reduced pore size can inhibit the release speed, but larger pores can accelerate it. The drug's ability to diffuse through the porous structure can be greatly affected by its solubility, molecular weight, and chemical composition. The release mechanism is influenced by the material composition of the nanoporous carrier itself.^59,60^

#### 2.2.2. Drug adsorption mechanisms

Drugs adhere to the surface of nanoporous substances because of weak forces like van der Waals or hydrogen bonds. This procedure is usually able to be reversible and is influenced by the material's surface area and characteristics. It involves the creation of more powerful chemical connections between drug compounds and the functional groups on NPMs' surface. Typically, this procedure leads to a stronger bond in comparison to physisorption. Adsorption isotherms, such as Langmuir and Freundlich, are employed to explain the adsorption of drugs onto material surfaces. These models have the ability to forecast the drug loading capacity of the nanoporous structure.^53,61^

A higher surface area and pore volume typically result in increased drug adsorption capacity. NPMs that have clearly defined pores have the potential to improve loading effectiveness. The adsorption affinity can be greatly affected by the functional groups on NPMs' surface.^18,62–64^ Higher initial drug concentrations in the solution tend to result in a higher adsorption capacity of NPMs, leading to increased loading. NPMs are commonly utilized to create precise drug release patterns, enhancing treatment effectiveness and reducing adverse reactions.

By altering the surface of nanoporous carriers, they can be created with a focus on delivering drugs to specific locations, such as tumor sites, to ensure targeted release of therapeutic agents. NPMs have the ability to co-deliver multiple drugs, improving the effectiveness of treatments for cancer and other illnesses. Understanding how drugs interact with NPMs through adsorption and release mechanisms is crucial for developing and using DDSs.

### 2.3. Biological characteristics

The biological characteristics of NPMs play a vital role in their biomedical applications, particularly in tissue engineering, drug delivery, and biosensing. These qualities dictate the way these substances engage with biological systems, affecting their effectiveness and safety. Listed below are primary biological traits of nanoporous substances:

Biocompatibility is the capacity of a material to interact with biological systems without eliciting a significant adverse immune response.^65^ Biocompatible NPMs are essential for applications like DDSs and tissue engineering scaffolds.^66,67^ They can reduce inflammation or anti-inflammatory responses and enhance cell survival.^68,69^ The surface chemistry of NPMs can be modified to enhance bioactivity, promoting favorable interactions with cells and biomolecules.^70–72^ Incorporating bioactive molecules, such as peptides or proteins, can enhance cell adhesion, proliferation, and differentiation, making the materials suitable for tissue engineering. Pores in NPMs directly affect cell adhesion, migration, and growth based on their size, shape, and distribution.^73–75^

In applications such as localized treatment of diseases, the capability to sustain and control drug release rates is essential.^22^ NPMs should degrade at a rate that matches tissue regeneration in many biomedical applications, especially in tissue engineering, ensuring gradual replacement by natural tissue.^76,77^ Some nanoporous carriers are engineered to degrade gradually (biodegradable polymers).^78^ The degradation process can be designed to control the rate at which the drug is released.^79–81^ The degradation products should be non-toxic and readily eliminated by the body, enhancing the safety profile of the materials.

NPMs can be used for their inherent antibacterial properties or to release antimicrobial agents, making them suitable to be used for wound healing and implants.^82–85^ Coating NPMs with antimicrobial agents can further enhance their ability to prevent infections.^86,87^ NPMs interacting with biological systems can elicit immune responses.^88,89^

NPMs can be tailored for precise biomolecule interactions, enhancing their effectiveness in biological applications.^71^ The porous structure enables the immobilization of enzymes for biocatalytic applications, offering improved stability and reusability.^70,90–92^ Functionalizing NPMs with targeting molecules (like antibodies or ligands) can enhance specificity in drug delivery. This enables targeted therapy in cancer treatment or other localized illnesses.^93,94^ Integrating signaling molecules to influence cellular responses contributes to designing more effective materials able to modulate biological responses.

The performance of NPMs in biomedical applications relies heavily on their biological characteristics. Their compatibility with living organisms, their ability to interact with biological systems, and their capacity to regulate drug release and interact with tissues directly affect their efficiency in medical applications such as drug delivery, tissue engineering, biosensing, *etc*.

## 3. Biosensing

Biosensing can be defined as the process of detecting a target molecule by mimicking biological systems such as the immune system. The presence of the detected target molecule can be identified by various methods of signaling, *i.e.*, electrochemical, optical, and photoelectrochemical. The principles upon which the active layer of a biosensor (the chemically sensitive layer) works can be classified as (1) bioaffinity, (2) catalytic, (3) transmembrane, and (4) cell sensors.^95,96^

### 3.1. Electrochemical biosensors

As the biosensor sizes decrease into the micro- and nano-scale ranges, the noise limitations of biosensing become more significant. Signal-to-noise ratio degradation becomes more obvious as the electrodes miniaturize. The use of nanostructured electrodes can improve the electrochemical signals, as reported in many studies.^97^ For example, Fu *et al.* showed that microporous gold electrodes can increase the noise limitations and lower the limit of detection (LOD) by fourfold in electrochemical biosensors. They also observed a 24-fold increase in signal levels. Using these nano-structured gold electrodes can result in higher sensitivity in almost every electrochemical aptamer sensor, including biosensors.^98^ Inducing binding to target molecules can be achieved by increasing active centers, which can in turn increase the charge mobility on the electrode surface. In another study, Emran and colleagues designed phosphorus-doped microporous carbon spheroidal structures (P-MCSs) with nanoporous carbon as a sensory electrode for selective adrenaline signaling in human fluids. This metal-free electrode structure has shown a very low LOD (0.002 µM) with ultra-sensitivity to its target molecule, adrenaline.^99^

MicroRNA expression can be dysregulated within cancer cells; therefore, it can be used as a biomarker for early stages of cancer diagnosis.^100^ Biosensors can provide a reliable and rapid method for detecting this biomarker. For instance, Xu and coworkers investigated a three-dimensional (3D) platinum nanoporous gold electrode as an ultrasensitive and low-cost sensor for detecting microRNA-126. They also used a catalytic hairpin assembly (CHA) reaction in combination with the platinum nanoparticles/nanoporous gold (PtNPs/NPG) electrode to achieve an improved electrocatalytic activity and subsequently a low background signal. They also observed a linear range of 0.5–500 pm and a LOD of 0.17 pm along with a high signal-to-noise ratio (S/N = 3).^101^

**Fig. 1:**
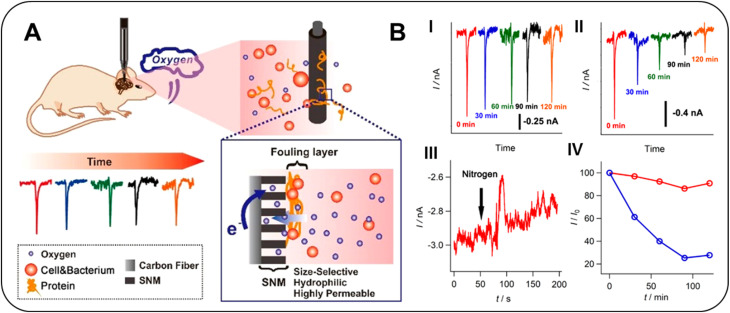
Utilization of an SNM as an antifouling barrier on carbon fiber electrodes for monitoring O_2_ within the rat brain: (A) diagram illustrating the hydrophilic, highly permeable SNM-coated CFME intended for ongoing O_2_ measurement. (B) (I and II) Amperometric responses from the SNM/c-CFME (I) and control c-CFME (II) implanted within the rat hippocampus over a 2 hour timeframe, with the rat receiving 7 seconds of pure O_2_ every 30 minutes, (III) amperometric response of SNM/c-CFME during N_2_ administration, and (IV) changes in normalized current (*I*/*I*_0_) over time for c-CFME (blue) and SNM/c-CFME (red) during the implantation period. This figure has been adapted/reproduced from ref. 102 with permission from the American Chemical Society, Copyright 2019.

Moreover, Zhou and colleagues developed a biosensor using a silica nanoporous membrane (SNM) coated on carbon fiber microelectrodes (CFMEs) to enhance the stability and sensitivity of *in vivo* oxygen (O_2_) monitoring in the rat brain. The nanoporous structure prevents biofouling while maintaining permeability to O_2_, allowing continuous and reliable monitoring for up to 2 hours (Fig. 1).^102^

**Fig. 2:**
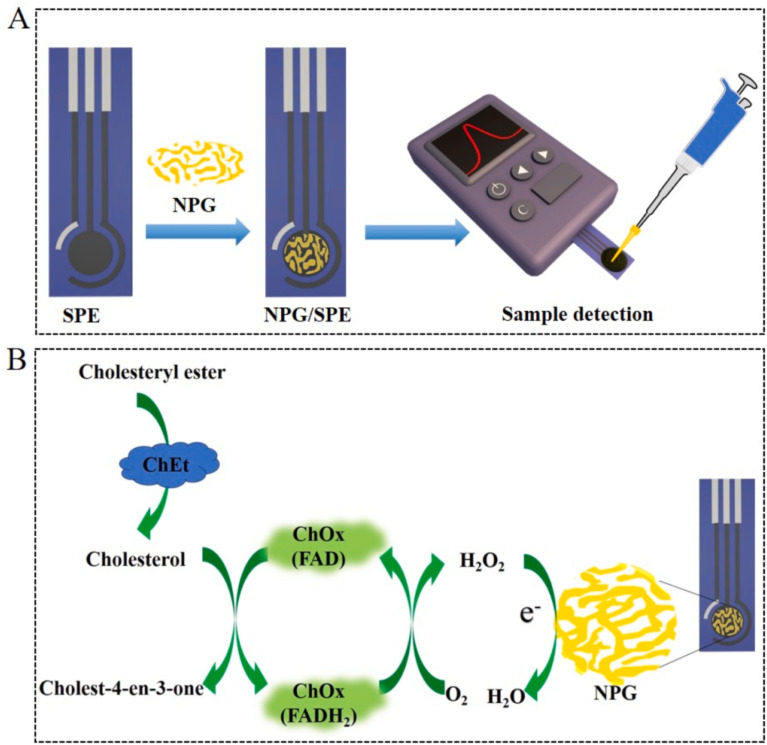
NPG/SPE-based biosensors for electrochemical detection of cholesterol. (A) Preparation of the NPG/SPE test strip and potential hand-held sample detection device. (B) The pathway of the detection strategy used in the biosensor. This figure has been adapted/reproduced from ref. 103 with permission from Elsevier, Copyright 2021.

Designing a convenient and cost-efficient biosensor for detecting cholesterol levels in blood can be useful for diagnosing and even preventing many cholesterol-related diseases, such as coronary heart disease and diabetes. For example, Wang *et al.* integrated nanoporous gold (NPG) with a screen-printed electrode (SPE) to design a biosensor with high sensitivity (32.68 µA mM^−1^ cm^−2^) and a wide linear range (50 µM–6 mM). In this sensor, cholesterol is oxidized to cholestene-3-one by cholesterol oxidase (ChOx), and H_2_O_2_ is produced in this process. H_2_O_2_ is then reduced to H_2_O by the catalytic activity of NPG. This process is detailed in Fig. 2, showing both the preparation of the NPG/SPE test strip and the pathway of the detection strategy.^103^

Using a similar strategy, Lin and coworkers immobilized the lactate oxidase enzyme on their graphene oxide (GO) nanoporous sensor for detecting lactate in human sweat. Lactate in the presence of lactate oxidase oxidizes to pyruvate and H_2_O_2_. Subsequently, H_2_O_2_ oxidizes, and hydrogen ions and electrons are produced in the process. The LOD and dynamic range of the sensor were reported as 1 mM and 1–100 mM, respectively.^104^

Developing a high surface area is a key goal of designing a porous structure for use in biosensors. Increasing the surface-to-volume ratio can lower the LOD and also increase the sensitivity of the censor. For example, Regiart *et al.* developed an ultrasensitive immunosensor with a gold nanoporous structure (NPAu) for the detection of SOX-2 cancer biomarkers. The SOX-2 antibody was immobilized in a microfluidic channel, and the nano-porous gold electrode was used as a detector. After introducing the sample to the sensor, SOX-2 molecules will bind to anti-SOX-2. As the next step, anti-SOX-2 conjugated with horseradish peroxidase is used for labeling SOX-2 present in the sample. At this point, adding H_2_O_2_ will cause a reduction reaction on the NPAu electrode, thereby resulting in a measurable current in the µA range. The detection limit of the sensor was reported as 30 pg mL^−1^.^105^

**Fig. 3:**
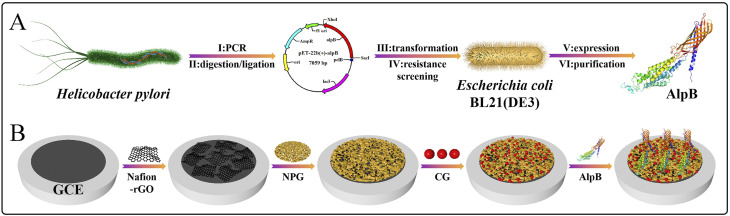
Detection and intensity measurement of six anti-biofilm drugs using the AlpB/CG/NPG/Nafion-rGO/GCE biosensor. (A) Expression of AlpB in the *E. coli* BL21(DE3) host. (B) Preparation steps of the AlpB/CG/NPG/Nafion-rGO/GCE biosensor. This figure has been adapted/reproduced from ref. 106 with permission from Elsevier, Copyright 2022.

The uses of biosensors are not limited to the detection of biomarkers and biological byproducts. Developing a sensitive and reliable protein-based sensor can be useful for drug discovery and screening and can be used as additional *in vitro* data alongside *in silico* methods such as molecular docking. In an interesting study, Xiao and coworkers developed an innovative biosensor composed of AlpB, nanoporous gold (NPG), Nafion-reduced graphene oxide (rGO), colloidal gold (CG), and a glassy carbon electrode (GCE) that can be used for screening potential drugs against *Helicobacter pylori* biofilms. The NPG component, characterized by its three-dimensional porous structure with nanoscale pores, provides a high surface area that enhances the biosensor's sensitivity by facilitating efficient immobilization of AlpB and improving electron transfer. AlpB, an outer membrane protein of *H. pylori*, plays an important role in the formation of biofilms. They identified 6 drugs that can be used against biofilm formation. In order of sensitivity, the six drugs were: allicin, erythromycin, SCC, curcumin, rifampicin, and NAC. Further kinetic analysis revealed the order of action intensity to be rifampicin, NAC, allicin, erythromycin, SCC, and curcumin, indicating a distinction between the drugs' potency and their apparent effectiveness in inhibiting biofilm formation. Fig. 3 illustrates the expression of AlpB in *Escherichia coli* BL21(DE3) and the biosensor's preparation steps using CG/NPG/Nafion-rGO/GCE.^106^

### 3.2. Optical biosensors

As discussed in the last section, electrochemical biosensing is achieved by measuring electrons with the use of electrodes and generating either a current or a voltage-based signal. Optical biosensing, on the other hand, uses photons as the source of information, and signaling can occur based on different interactional properties of light, such as absorbance, reflectance, or emission. These changes can then be detected by a light sensor or even a simple smartphone-based device, as will be discussed in the following section.^107^

**Fig. 4:**
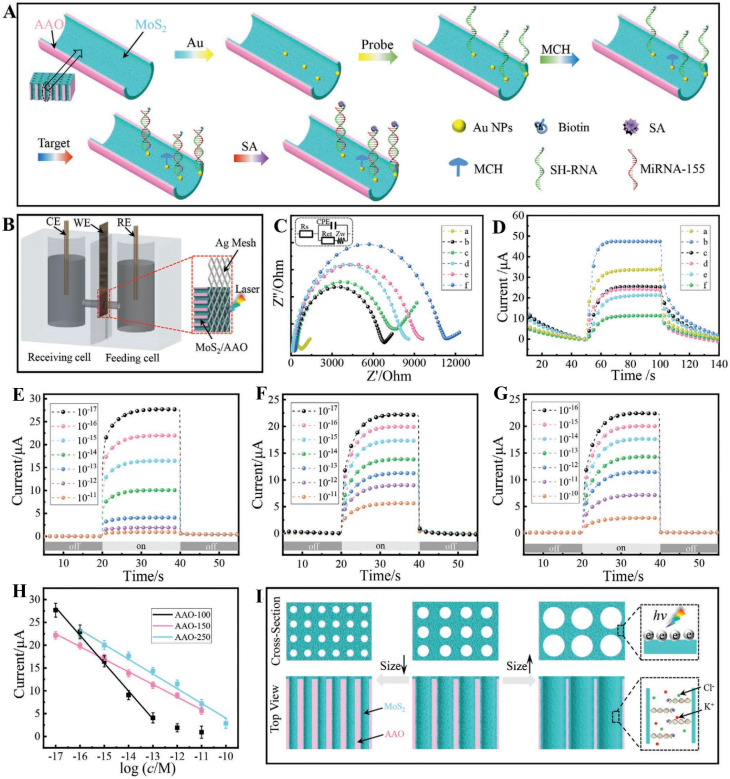
Biosensing application of nanoporous membranes. (A) Fabrication procedures of the miRNA detection principle using MoS_2_-modified AAO nanochannels with gold nanoparticles (AuNPs) and a biotin-labeled SH-RNA probe for hybridization with target miRNA-155, followed by streptavidin (SA) binding; (B) diagram of a photoelectrochemical cell featuring a porous silver mesh electrode connected to the MoS_2_/AAO nanochannels for detecting miRNA-155, facilitating ionic transmission; (C) electrochemical impedance spectroscopy (EIS) at various fabrication stages; (D) photocurrent response data were obtained at various fabrication stages; (E–G) photocurrent responses to varying concentrations of miRNA-155 for AAO nanochannels with diameters of 100, 150, and 250 nm; (H) calibration curve of current *versus* logarithm of miRNA-155 concentration; (I) mechanism illustrating the effect of nanochannel size on biosensing performance; the AAO membrane thickness is approximately 95 µm. This figure has been adapted/reproduced from ref. 110 with permission from Wiley-VCH, Copyright 2020.

Cathepsin B (Cat B) – a lysosomal cysteine protease – can be used as a cancer biomarker, as many researchers suggest. The CTSB gene is regulated under normal conditions, but overexpression of Cat B is reported to be correlated with malignancy and metastatic cancers.^108^ In a study, Amouzadeh Tabrizi and colleagues designed an interferometric biosensor with modified nanoporous anodic alumina (NAA) that can detect the presence of the said biomarker in human serum. They immobilized human serum albumin (HSA) into the pores of the NAA and then labeled the HSA with thionine (HSA-TH). HSA-TH is digested in the presence of Cat B, and short peptide fragments are released. This process causes changes in the optical properties of the biosensor and also increases the light reflected to the detector. They reported a LOD of 0.08 nM.^109^ Biosensing platforms have benefited greatly from the application of nanoporous membranes, as shown in Fig. 4. To illustrate how pore size affects biosensing efficiency, miRNA-155 has been detected using photoelectrochemical biosensing on MoS_2_-modified porous anodic aluminum oxide (AAO) nanochannels functionalized with streptavidin (SA). The optimal nanochannel diameter of about 130 nm (named AAO-150) provided the lowest detection limit of 3 aM due to a balance between photogenerated current (which decreases with larger pores due to the reduced MoS_2_ deposition area) and ion transmission (which is hindered more in smaller pores by bound biomolecules).^110^

The advantages of using nanoporous structures in biosensors are not limited to their low LOD and high sensitivity. Nanoporous microneedles can be used as both sample collection and detection methods for a painless, ultrasensitive biosensing approach. For instance, Kang and colleagues designed an immunoassay-based biosensor with the use of nanoporous microneedle (nMN) structures that can capture estrogen (E2) and, after a short incubation time (1 minute), measure E2 levels ranging from 0.5 ng mL^−1^ to 1000 ng mL^−1^. They immobilized anti-E2 antibodies in the pores of the microneedles. After capturing the E2 present in the subcutaneous interstitial fluids (ISFs), microneedles are removed, and a second fluorescent-labeled anti-E2 is presented to the structure. The E2 measurements can then be performed using a fluorescence microscope.^111^

One of the limitations of optical and electrochemiluminescence (ECL) biosensing in particular is the requirement of auxiliary equipment, which is often large in size, for light detection and other processes that need to take place after the sample collection step. A handheld ECL device was designed by Zhu and coworkers, which takes advantage of a smartphone with an Android application for easy and user-friendly interaction with the device. They also designed an indium tin oxide glass electrode coated with antifouling SNM to calibrate and test the device by detecting dopamine (DA). A linear range of 5 nM–20 µM was reported for this electrode, and the LOD was 3.5 nM. In addition, for mimicking clinical conditions and testing real-life bio-samples, urine and rat brain homogenate were used as samples. Rat brains were stimulated with high levels of potassium beforehand, and the ECL device detected an increase in the level of dopamine in these samples.^112^

The COVID-19 pandemic nearly exceeded the limits of today's healthcare and medical capabilities. As of May 17, 2023, it is considered one of the deadliest pandemics in history, with an estimated 7 million deaths reported to the WHO.^113^ With vaccination, lockdowns, and public hygiene measures, new cases are lower, and many countries no longer have restricted laws on masks or social distancing. At that time, more than 13.5 billion vaccine doses had been administered (WHO Coronavirus Dashboard).^114^ Developing a fast, reliable, and low-cost method for measuring the immunity caused by vaccines can be useful for future prevention and even ending the COVID-19 pandemic. For instance, Huang *et al.* developed a nanoplasmonic immunosorbent assay (NanoPISA) platform for such a purpose. They used nanoporous hollow gold in the sensor and achieved a LOD of 0.2 pM for quantitatively detecting the neutralizing antibodies (NAs) specific to SARS-CoV-2 spike protein within samples of human serum.^115^Table 1 summarizes applications of nanoporous materials and related composites for sensing.

**Table 1:** Sensing applications of nanoporous materials and their composites^a^

Nanomaterials	Composite	Sensor type	Target molecule/cell	LOD	Sensitivity	Ref.
Au	AuNP-PEDOT/GCE	Electrochemical	GSH	0.173 µM	10.7 µA cm^2^ µM^−1^	116
Au	6-FcHT/NPGL/GE	Electrochemical	Hydroxyl radicals	0.133 nM	0.0305 mA nM^−1^	117
Au	Au/3D-CS/rGO/GCE	Electrochemical aptasensor	Acetamiprid residues	71.2 fM	N. A.	118
Au	NPGL	Electrochemical	Organophosphates (paraoxon)	0.53 pM	376 nA nM^−1^	119
Fe_3_O_4_	Fe_3_O_4_/biotin–streptavidin	Electrochemical immunosensor	Zearalenone	3.7 pg mL^−1^	10.0 pg mL^−1^–12.0 ng mL^−1^	120
NAA	NAA functionalized with APTES and glutaraldehyde	Label-free biosensor based on RIFTS	hGH	0.6 µg mL^−1^	0.21 nm µg^−1^ mL	121
Anodic alumina	NAA membrane	Electrochemical	Flightless I protein	1.04 µg mL^−1^ (lowest)	33.69 mL µg^−1^ (highest)	122
Anodic aluminum oxide	DAN/AAO	Electrochemical	Circulating tumor cells	80 cells per mL	N. A.	123
NPGCE	PEI/TetX_2_/PTH/NPGCE	Optical/colorimetry	Tetracycline	60 nM	N. A.	124
Nanoporous silica	Silica gel matrix on puuE	Optical/fluorescence	Allantoin	N. A.	N. A.	125
TiO_2_	Ti/TiO_2_-Pb_5_S_2_I_6_-PDA	Optical/PEC	Cr(vi) ions	3.0 nM	N. A.	126
TiO_2_	Ordered porous PS–TiO_2_ composite	Continuous flow biosensor	SPA and hIgG	N. A.	N. A.	127

a Abbreviations: APTES, aminopropyltriethoxysilane; AAO, anodic aluminum oxide; CS, chitosan; DAN, dendrimer-AuNP network; 6-FcHT, 6-(ferrocenyl) hexanethiol; GCE: glassy carbon electrode; GSH, glutathione; GE, gold electrode; hGH, human growth hormone; hIgG, human serum immunoglobulin G; NAA, nanoporous anodic alumina; NPGCE, nanoporous glassy carbon electrode; NPGL, nanoporous gold layer; PEDOT, PEC, photoelectrochemical; PDA, polydopamine; poly(3,4)ethylene dioxythiophene; PEI, polyethyleneimine; PS, polystyrene; rGO, reduced graphene oxide; RIFTS, reflective interferometric Fourier transform spectroscopy; SPA, *Staphylococcus aureus* protein A.

## 4. Medical applications

### 4.1. Tissue engineering

#### 4.1.1. Importance of porosity in scaffold design

Porosity is a crucial aspect of scaffold design in tissue engineering, as it directly influences cellular behaviors like adhesion, migration, and proliferation while also supporting tissue formation. A well-designed scaffold, with the right combination of pore size, distribution, and connectivity, facilitates cell–scaffold interactions, ensures nutrient delivery, and allows for effective waste removal, all of which are essential for successful tissue regeneration.^128^

Scaffolds typically feature a range of pore sizes: macropores (100–500 µm) support cell attachment, tissue growth, and blood vessel formation, and smaller pores in the micrometer range (<100 µm) and nanometer range (<100 nm) aid in delivering biomolecules and increasing the scaffold's surface area for improved cell interaction. These complex structures can be achieved through various fabrication methods, including freeze-drying, templating, or 3D printing.^129^

The optimal pore size depends on the specific tissue being regenerated. For example, bone scaffolds perform best with pores around 325 µm, which promotes tissue growth and vascularization, whereas skin regeneration benefits from pore sizes between 20 and 125 µm.^112,130,131^ Interconnected pores larger than 100 µm facilitate cell infiltration and extracellular matrix (ECM) deposition, while pores in the micrometer range enhance cell ingrowth, attachment, and nutrient absorption.^132,133^

Nanopores, included in scaffold designs, can further enhance cell–material interactions by increasing the surface area and improving scaffold performance.^134^ Additionally, porous nanomembranes like anodic alumina can regulate the diffusion of molecules, making them promising candidates for drug delivery systems.^135^Table 2 summarizes applications of nanoporous materials and related composites for tissue engineering.

**Table 2:** Tissue engineering applications of nanoporous materials and their composites^a^

Composition	Nanostructure	Cell/tissue	Synthesis method	Critical analysis	Pore size	Significant outcome	Ref.
Hydroxyapatite (HAP)-based scaffold	Nanocomposite	Mouse preosteoblast (MC3T3-E1) cells/bone	Synthesis of HAP NPs, pressing, and low-temperature sintering	FTIR, SEM, TEM, XRD, MIP, DLS, compression tests, MTT and ALP assays, confocal laser microscopy, local lymph node assay	16.5–30.2 nm	The optimal nanoporous size (30.2 nm) significantly increased mechanical strength and enhanced cell proliferation and differentiation rates, leading to better bone regeneration efficiency *in vivo* compared to a commercial bone graft material	20
SF sponges with walls containing nanopores (SFNS)	Sponge walls	rMSCs	Autoclaving the SF solution to generate SF nanoparticles, followed by freeze-drying and leaching the SF nanoparticles from the walls	SEM, FTIR, XRD, BET/BJH, water absorption, porosity measurement, LSCM, AlamarBlue assay, H&E staining, and immunofluorescence	Macropores (100–200 µm)	SFNS had significantly increased porosity and high biofluid-absorbing capacity (over 30 times its weight), resulting in better rMSC proliferation and superior acceleration of wound closure and angiogenesis *in vivo* compared to sponges without nanopores	22
					Nanopores (50–300 nm)		
SF/GE blend polymeric matrix	Nanofibrous structure	Statens Seruminstitut rabbit cornea cells (SIRC)/corneal epithelial TE	Electrospinning, followed by cross-linking using EDC-NHS	Viscometry, SEM, ATR-FTIR, transparency measurements, DSC, tensile test, porosimetry, ROS evaluation (confocal microscopy), MTT assay, degradation test	175–267 nm	The pore size of the SF/GE scaffolds was found to be inversely proportional to the gelatin concentration. The SF/GE 50 : 50 blend was identified as the most potential scaffold matrix for CTE	136
PCL-PEG-PCL (PCEC) triblock copolymer scaffold loaded with DXMT	The scaffold	N. A.	ROP and scCO_2_ gas foaming	BET/BJH, mercury porosimetry, helium pycnometry, XRD, DSC, UV-vis spectrophotometry, SEM, compression tests, CCD using RSM	84.69 ± 5.36 nm	Drug release and scaffold degradation are directly related to each other and inversely related to pore size. When exposed to scCO_2_, the scaffold's pore size decreased while its porosity increased. This change in structure accelerated both the DXMT release and scaffold degradation	137
Furfuryl-gelatin (f-gelatin) and PCL	Nanofibrous scaffold	Human cardiomyocyte cells (AC16) and Cellartis human iPSC derived cardiomyocytes/cardiac TE	Single nozzle electrospinning or coaxial electrospinning, followed by visible light cross-linking	SEM, TEM, TGA, DSC, FTIR, FACS, rheological analysis, and confocal microscopy	N. A.	Although the source does not directly analyze the effects of specific pore sizes, the findings suggest that controlling the swelling behavior of f-gelatin, through blending with PCL, is crucial for maintaining appropriate pore structure and promoting cell integration within the scaffold	138
PCL/GE/om-CMS nanocomposite scaffold	om-CMS particles	Osteoblast-like cells (MG-63)/bone	3D printing (extrusion-based 3D printer)	TEM, SEM, EDS/map, XRD/LAXRD, FTIR/FTIR-ATR, BET/BJH, *in vitro* biodegradation, compression tests, MTT and ALP assay, DAPI staining, ICP	om-CMS: 3.09 nm	Ceramic content significantly improved the material properties, increasing compressive strength, hydrophilicity, bioactivity, cell proliferation, and alkaline phosphatase activity, while reducing the degradation rate	139
					Scaffolds: 400–500 µm		
PCL sheath and PEO core	Nanoporous microtubes	N. A./vascularization and capillary formation	Core-sheath electrospinning and phase separation for surface nanopore formation	Viscometry, porosimetry, SEM, and ANOVA statistical analysis	138–252 nm	Higher solution viscosity significantly increased microtube diameter and pore size. The addition of PEO into the PCL sheath also facilitated surface nanopore formation. The optimal parameters (high viscosity, PCL/PEO ratio of 3 : 1) resulted in the highest average microtube diameter and pore size (252 nm), mimicking native capillaries	140
PLA scaffold	Hierarchical nanocomposite	K562 cells/bone TE	Fused filament fabrication (FFF) 3D printing and supercritical CO_2_ batch gas foaming	RSM, SEM, mechanical assessment, MTT assay	Nanoporous (<400 nm) and porous structure (∼500 µm)	Pore size is significantly affected by gas foaming parameters. By carefully controlling the gas foaming parameters, researchers can create scaffolds with hierarchical pore structures that mimic natural bone tissue, enhance cellular adhesion, and exhibit excellent mechanical properties	141
PLA and PEO (sheath) and PEO (core)	Nanoporous microtube	GFP human dermal microvascular endothelial cells (HDMVE)/capillary vessels	Core–sheath electrospinning	SEM, digital rotational viscometry, fluorescence imaging	100–800 nm	Inconsistent viscosities of the core and sheath solutions resulted in the formation of larger nanopores within the resultant nanoporous microtubes. These structures were compatible with human endothelial cells and supported cell growth	142
PLA/COL hybrid fibrous membranes	Nanoporous aligned PLA fibers	SCs, DRG neurons, and L929 cells/peripheral nerves	Electrospinning; vapor-induced phase separation to form nanopores	FTIR, SEM, WCA, tensile test, MTT assay, hemolysis assay, EdU assay, and immunofluorescence	N. A.	Nontoxic and demonstrated good hemocompatibility; promoted SC proliferation and elongation, and enhanced the extension of DRG neurites	143
PLA/titanium dioxide (TiO_2_) nanocomposite membranes	Nanofibrous structure	Fibroblast cells (L929)/skin	Electrospinning	SEM, TEM, TGA, DSC, tensile test, XRD, MTT assay, RT-qPCR, histological analysis	N. A.	Non-cytotoxic; upregulated versicam (VCAN) and type-1 collagen (COL1A1) gene expression; stimulated blood vessel formation; demonstrated potential for reducing cutaneous scarring	144
NAA and macroporous silicon (MacroPSi) substrates, modified with collagen, and fibronectin	NAA	HAEC/vascularization	Anodic dissolution for MacroPSi and a two-step anodization process for NAA, followed by APTES and glutaraldehyde crosslinking for covalent protein binding	SEM, AFM, CFM	MacroPSi substrates: 1–1.2 µm	The cells exhibited significantly better adhesion, morphology, and proliferation on Fn-functionalized surfaces compared to Col; MacroPSi produced lamellipodia, while NAA resulted in thin filopodia, indicating pore size influenced cell morphology; cell proliferation was higher on NAA-Fn substrates, suggesting that proliferation is enhanced when the pore size is smaller	145
					NAA substrates: 30–40 nm		
PVA/maghemite	External nanoporous structure	Human fibroblast cells/skin	A processing technique combining fused deposition modelling (FDM) 3D printing, TIPS method, and electrospinning	FESEM, compression tests, degradation test, cell penetration study	N. A.	The 3D scaffold exhibited suitable mechanical properties for hard tissue engineering. Initial biocompatibility tests confirmed that the scaffold can support the growth and penetration of human fibroblast cells	146
Magnetic zinc silicate-ZnFe_2_O_4_ core–shell composite loaded with ibuprofen	Zinc silicate shell	Osteoblast-like cells (MG63)	A two-step strategy: solvothermal method for ZnFe_2_O_4_ particles and surfactant-assisted and sol–gel method for coating with zinc silicate	XRD, FESEM, VSM, EDX, BET/BJH, UV-vis spectroscopy, MTT assay, agar well disc diffusion (AWDD) method	1.64 nm	Offered a hyperthermia capability, reaching a saturation temperature of 45 °C, effective antibacterial activity, and controlled drug release for advanced bone tissue engineering applications	147
Laponite diffusion gels	Laponite aggregations	HBMSCs/skeletal tissue	Diffusion/dialysis gelation process	SEM, EDX/EDS, rheological analysis, PCR, immunocytochemistry, Alizarin red staining (ARS)	10–2000 nm	The gels enhanced cell-mediated matrix mineralization and demonstrated high cytocompatibility even at high concentrations. In 3D diffusion gels, HBMSCs exhibited extensive osteogenic matrix synthesis compared to scaffold-free controls	148
Tantalum samples with tantalum oxide surfaces	Ta_2_O_5_ surface	Fibroblast cells (L929)	Electrochemical anodization using aqueous HF:H_2_SO_4_ electrolyte under 30 V potential	SEM, TEM, AFM, XPS, BET, topography, MTT assay, fluorescence microscopy (actin/DAPI staining)	25–65 nm	Anodized nanoporous tantalum oxide surfaces enhanced fibroblast proliferation and cellular spreading, with the 25 nm pore size showing the best biological response	149
CS membranes with PEG as the porogen	CS membrane	Human keratinocytes/epidermal tissue	Phase inversion technique by evaporation-induced phase separation	Membrane surface properties affect keratinocyte stratification and differentiation, SEM, AFM, tensile testing, MTT, CFP	26 nm and 131 nm	Membrane surface porosity significantly modulated cellular differentiation. On mesoporous membranes (with a pore diameter of 26 nm), keratinocytes formed a thin corneum epidermal layer with low proliferation. On macroporous membranes (with a pore diameter of 131 nm), cells stratified and differentiated over time, forming a multilayered epidermis with high proliferation	150
PCMs	PCMs	hPDLCs/bone	N. A.	FTIR, AFM, SEM, EDS, water contact angle analysis, ARS, RNA sequencing, RT-qPCR, pathway analysis (GO and KEGG enrichment), confocal microscopy, migration assay, *in vivo* skull defect	200 nm, 800 nm, 1200 nm, and 10 µm	The 200 nm PCM demonstrated the optimal osteo-induction ability both *in vitro* (highest mineralization and ALP upregulation) and *in vivo* (accelerated healing in mice skull defects, proven ectopic osteogenesis). RNA sequencing indicated significant upregulation of osteogenic regulatory pathways, including Hippo, TGF-β, and PI3K-Akt signaling	151

a Abbreviations: ASCs, adipose tissue-derived stem cells; AMC, adriamycin; AFM, atomic force microscopy; ATR, attenuated total reflectance; BJH, Barrett–Joyner–Halenda; BTC, benzene-1,3,5-tricarboxylate; BET, Brunauer–Emmett–Teller; CFP, capillary flow porometry; CCD, central composite design; CS, chitosan; CHX, chlorhexidine; COL, collagen I; CFM, confocal fluorescence microscopy; CTE, corneal tissue engineering; COF, covalent organic framework; CUR, curcumin; DTG, derivative thermogravimetric; DXMT, dexamethasone; DSC, differential scanning calorimetry; DMOG, dimethyloxalylglycine; DRG, dorsal root ganglion; DOX, doxorubicin; EDS, energy dispersive spectroscopy; EDAX, energy dispersive X-ray spectroscopy; 5-FU, 5-fluorouracil; FTIR, Fourier-transform infrared; GE, gelatin; GIC, glass ionomer cement; HPLC, high-performance liquid chromatography; HAEC, human aortic endothelial cells; HBMSCs, human bone marrow stromal cells; HDMVE, human dermal microvascular endothelial cells; HOF, human oral fibroblast cells; HOK, human oral keratinocyte cell; hPDLCs, human periodontal ligament cells; HUVECs, human umbilical vein endothelial cells; HAP, hydroxyapatite; IBU, ibuprofen; ITZ, itraconazole; MIP, mercury intrusion porosimetry; MOFs, metal–organic frameworks; MCF-7, Michigan Cancer Foundation-7; NOA, nanoporous optical antenna; NPS, nanoporous silica; NPSNPs, nanoporous silica nanoparticles; om-CMS, ordered mesoporous calcium magnesium silicate; OMCs, ordered mesoporous carbons; PMO, periodic mesoporous organosilica; PAMAM, polyamidoamine; PCL, polycaprolactone; PCMs, polycarbonate membranes; PEG, polyethylene glycol; PEO, polyethylene oxide; PLA, polylactic acid; PVP, polyvinylpyrrolidone; PXRD, powder X-ray diffraction; QC, quercetin; rMSCs, rat mesenchymal stem cells; ROP, ring-opening polymerization; RNP, ribonucleoprotein complex; SEM, scanning electron microscopy; SCs, Schwann cells; SF, silk fibroin; scCO_2_, supercritical carbon dioxide; SERS, surface-enhanced Raman scattering; TC, tetracycline; TIPS, thermal inversion phase separation; TGA, thermogravimetric analysis; TEM, transmission electron microscopy; TMP, trimethoprim; VEGF, vascular endothelial growth factor; VSM, vibrating sample magnetometer; WCA, water contact angle; XRD, X-ray diffraction.

#### 4.1.2. Hard tissue engineering

Hard tissue engineering focuses on the regeneration and repair of rigid tissues such as bone and cartilage. It involves the use of biomaterials that provide mechanical strength and support to withstand physical stresses while facilitating the integration of new tissue growth. Scaffolds in hard tissue engineering often need to be porous for cell infiltration and vascularization, but also strong enough to support load-bearing functions. This field addresses issues like bone defects, fractures, and osteoarthritis through engineered implants and tissue substitutes.

Biocomposites have to be biodegradable, biocompatible, and stable to be utilized for applications including tissue engineering. Collagen is a biomaterial widely used in tissue engineering due to its remarkable biocompatibility. Nevertheless, pure collagen has not been suitable for bone tissue engineering because of its poor mechanical strength. So, fabricating a 3D-nanoporous structure with the desired mechanical strength has been a challenge. To address this, a low-temperature 3D printing process was employed to fabricate a nanoporous hybrid scaffold. This scaffold incorporates a decellularized extracellular matrix (dECM) and collagen to promote cellular activity and SF to enhance mechanical strength. *In vitro* assessment using MC3T3-E1 pre-osteoblast cells compared the performance of pure collagen, collagen/dECM (CE), and collagen/dECM/SF (CES) scaffolds *via* MTT assay, cell viability, and osteogenic activity analyses. Results showed significantly improved cell proliferation, differentiation, and viability on the CE and CES scaffolds compared to pure collagen, demonstrating the efficacy of the hybrid approach for bone tissue engineering applications. According to these results, incorporating dECM can significantly improve the cytoskeleton. This improvement is attributed to the dECM's bioactive components, including peptides, polysaccharides, and growth factors. Furthermore, its inherent 3D structures can promote cell adhesion, migration, proliferation, and differentiation. Overall, CES scaffolds have proven to be highly effective for hard tissue regeneration.^152^

**Fig. 5:**
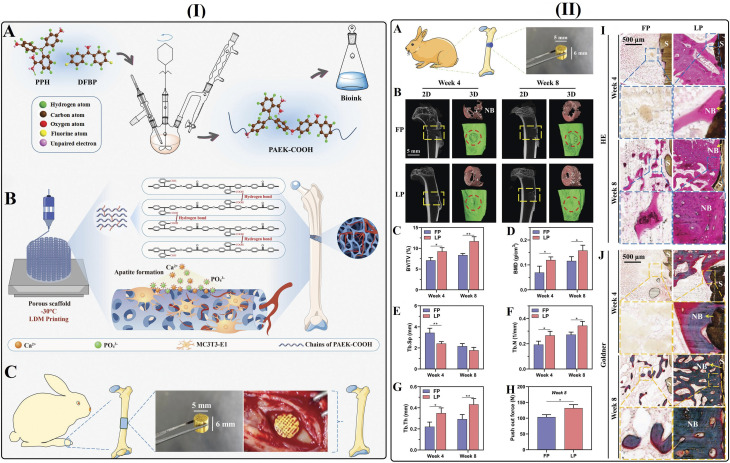
(I) Schematic representation of hierarchically porous PAEK-COOH scaffold fabrication and its role in bone regeneration. (I-A) PAEK-COOH synthesis and bioink preparation (PPH: phenolphthalein; DFBP: 4,4′-difluorobenzophenone). (I-B) Hierarchical porous structure of LDM-printed PAEK-COOH supporting cell adhesion and mineralization. (I-C) *In vivo* bone formation stimulated by the implanted scaffold. (II-A) Schematic of scaffold implantation process (size: 5 mm diameter, 6 mm depth). (II-B) 2D/3D micro-CT images of regenerated bone (red, NB: new bone) around defects with FP and LP scaffolds at 4 and 8 weeks. (II-(C–G)) Quantitative metrics for regenerated bone. (II-H) Push-out force of scaffolds at 8 weeks. (II-I and J) Representative HE (I) and Goldner (J) staining of bone repair (S: scaffold, NB: new bone; yellow arrows: new bone along scaffold). This figure has been adapted/reproduced from ref. 153 with permission from Wiley-VCH, Copyright 2022.

A hierarchically porous-structured polyaryletherketone (PAEK) is usually fabricated to improve osteogenic activity, but high-temperature fabrication methods limit its application. In one study, a low-temperature 3D-printing process was employed to fabricate a hierarchically porous PAEK scaffold modified with carboxyl groups (PAEK-COOH) with pore sizes ranging from 0.2 to 3.0 µm at the nanoscale and hundreds of microns at the microscale (Fig. 5).^153^ PAEK is generally used in clinical orthopedic applications because of its mechanical properties, biocompatibility, chemical resistance, and similar elastic modulus to natural bone. An *in vitro* study showed that electrostatic interactions of carboxyl groups can result in hydroxyapatite mineralization, accelerating bone formation. Two different scaffolds were fabricated by low-temperature deposition modeling (LDM) and fused deposition modeling (FDM). Then, LP (LDM-printed) and FP (FDM-printed) scaffolds were examined to compare their effects on cellular behaviors, with emphasis on the role of porosity. Cells cultured on nanoporous scaffolds (LP) showed increased cellular adhesion, spreading, and osteogenic differentiation compared to cells on nonporous scaffolds (FP), suggesting that nanoporous scaffolds potentially induce cellular behavioral enhancement and improve osteogenic capacity. After 14 days, LP scaffolds showed significantly higher alkaline phosphatase (ALP) activity than FP scaffolds. Alizarin Red S (ARS) staining at 21 days revealed greater calcium deposition on LP scaffolds. Furthermore, LP scaffolds exhibited upregulated expression of osteogenic genes, including osteocalcin (OCN) and ALP. *In vivo* experiments demonstrated that the LP scaffolds offered much better osseointegration without additional active ingredients, compared to FP ones, as shown in the schematic of the scaffold implantation process and the 2D and 3D reconstruction micro-CT images of regenerating bone around defects where LP and FP scaffolds were implanted (Fig. 5-II). These results indicated that LDM-fabricated PAEK-COOH scaffolds promoted superior bone tissue formation and integration at the defect site compared to FDM-fabricated scaffolds.^153^

Among the therapies for bone defect treatment, tissue engineering is considered the ideal one. In one study, micro/nanoporous polycarbonate membranes (PCMs) have been used as ECMs for culturing human periodontal ligament cells (hPDLCs) and studying their osteogenic differentiation. Likewise, PCMs with pore diameters of 200 nm, 800 nm, 1200 nm, and 10 µm were compared to identify their physical properties. *In vivo* experiments indicate that stimulation of hPDLCs with 200 nm PCMs significantly enhanced bone regeneration. An investigation of the regulatory system was conducted employing RNA sequencing, gene ontology analysis, and Kyoto Encyclopedia of Genes and Genomes (KEGG) pathway analysis. The results revealed a significant upregulation of the osteogenic markers Hippo, TGF-β, and PI3K-Akt. The observed upregulation of osteogenic markers highlights the potential of nanoporous PCMs for bone regeneration. *In vitro* studies using ALP and Alizarin Red staining demonstrated that the 200 nm PCMs exhibited the most promising results, showing denser ALP staining after 14 days and greater ECM mineralization after 21 days compared to other PCM sizes. These findings support the development of novel strategies to enhance hPDLC osteogenesis and bone tissue engineering.^151^

#### 4.1.3. Soft tissue engineering

Soft tissue engineering applies principles from biology, materials science, and engineering to develop solutions for repairing and regenerating soft tissues like skin, muscle, tendons, and ligaments. The goal is to create biomaterials and scaffolds that mimic the native tissue environment to support cell growth and restore the tissue's structural and functional roles. Applications include wound healing, skin regeneration, cardiovascular tissue repair, and musculoskeletal restoration. Challenges include mimicking complex tissue structure, achieving biocompatibility, and promoting vascularization.

Fabrication of vascular networks is a major challenge in complex tissue development. The existing artificial vascular channels are too large (millimeter or submillimeter scale), while human capillaries are only 5–10 µm in diameter. According to a study, during a core-sheath electrospinning procedure, nanoporous microtubes were fabricated to simulate a capillary structure. The composite of PLA and PEG sheath solution, with PEG as the core, produced microtubes (0.9–7.6 µm diameter) containing nanopores (130–820 nm). These nanoporous microtubes greatly resemble human capillaries. To study the cell attachment to the microtubes, experiments were performed using human dermal microvascular endothelial cells that express green fluorescent protein (GFP). Microscopy revealed cell attachment after 24 hours and a random distribution across the microtubes after 3 days, demonstrating the biocompatibility of these electrospun microtubes with endothelial cells. These nanoporous microtubes hold promise for enhancing vascularization in tissue engineering scaffolds.^154^

**Fig. 6:**
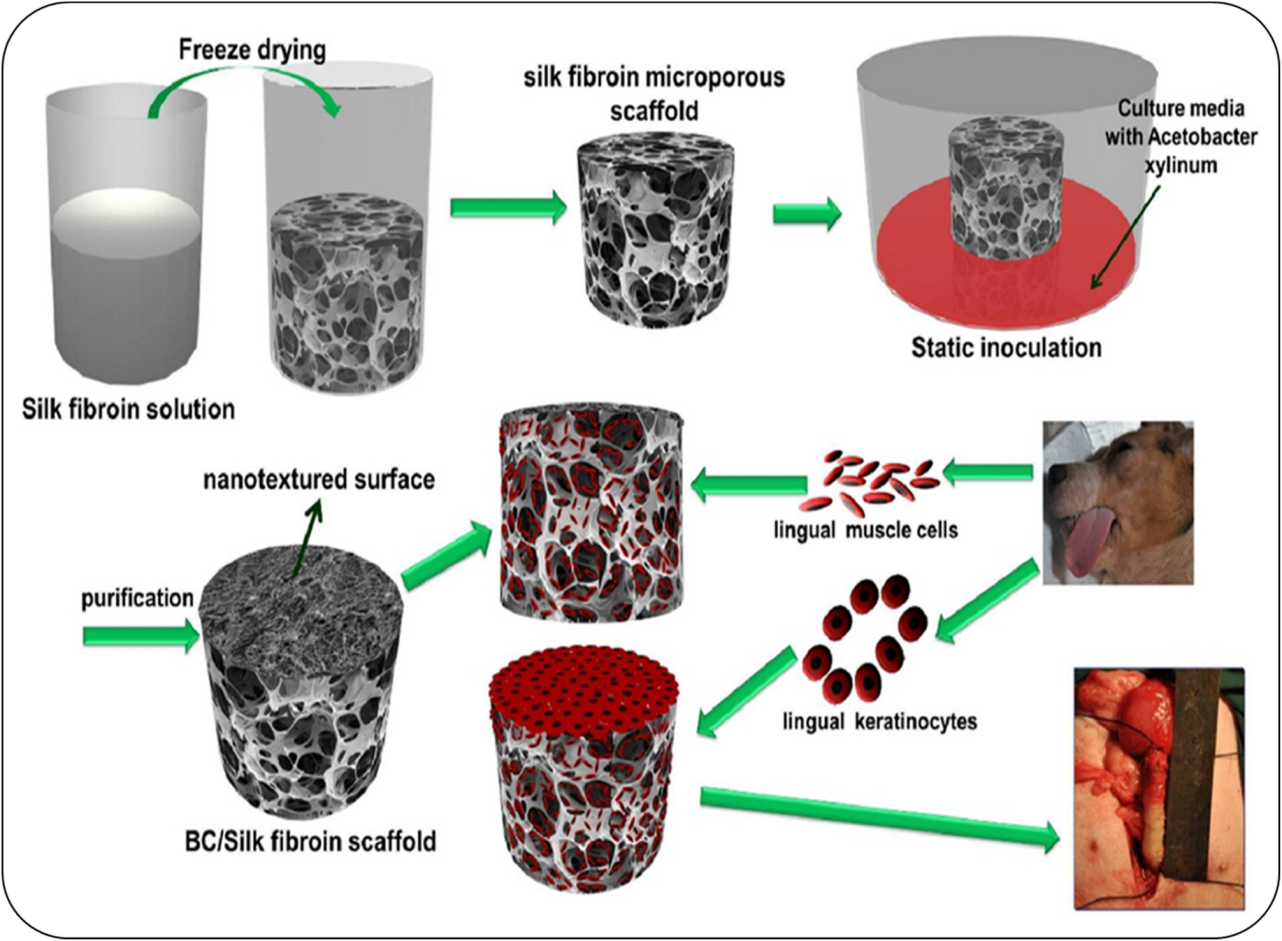
Schematic illustrating the biosynthesis of bilayer SF-BC composites and their application in fabricating a tissue-engineered urethra. This figure has been adapted/reproduced from ref. 155 with permission from Ivyspring International Publisher, Copyright 2018.

The limited success of current urethral tissue engineering methods stems from inadequate scaffolds that fail to support diverse cell types. To address this, a novel bilayer scaffold, composed of nanoporous bacterial cellulose (BC) and a microporous SF network using freeze-drying and self-assembly. The fabrication of a tissue-engineered urethra using a bilayer SF-BC composite is schematically illustrated in Fig. 6.^155^ Nanotextured BC in a bilayer scaffold promoted cell proliferation, while the microporous SF layer facilitated cell migration and integration with surrounding tissues. Scanning electron microscopy (SEM) and microcomputed tomography confirmed the scaffold's bilayer structure, 85% porosity, and BC nanofibers with an average diameter of 47 nm. Lingual keratinocytes and muscle cells seeded onto the scaffold demonstrated good cell adhesion and proliferation. MTT assays and cytotoxicity tests showed excellent biocompatibility, with optical density (OD) values comparable to those of the controls. In a canine urethral defect model, scaffolds seeded with cells exhibited superior structural integration after 3 months compared to unseeded controls. The high biocompatibility of this SF-BC scaffold suggests potential applications in various tissue engineering fields, including vascular, bladder, ureteral, bowel, and intestinal reconstruction.^155^

Skin tissue engineering has been a major challenge due to the difficulties of creating a partial or complete human epidermis. To achieve this goal, nanoporous and microporous chitosan (CS) membranes with specific surface properties were fabricated by the phase inversion technique, leveraging CS's biocompatibility, biodegradability, and antimicrobial properties. Culturing human keratinocytes on these membranes for 21 days revealed robust cell adhesion and the formation of a multilayered epidermis with high proliferation. SEM imaging confirmed a homogeneous, smooth membrane surface with 26 nm nanopores. Keratinocytes migrated along the *z*-axis, forming a basal lamina, and differentiation was evidenced by increased glucose consumption (peaking at 975 ± 35 µg mL^−1^ on day 14) and significant CK1 expression after 21 days. This novel approach provides a platform for evaluating the effects and toxicity of cosmetics, drugs, and pollutants using a reconstructed human epidermis.^156^

### 4.2. Wound healing

**Fig. 7:**
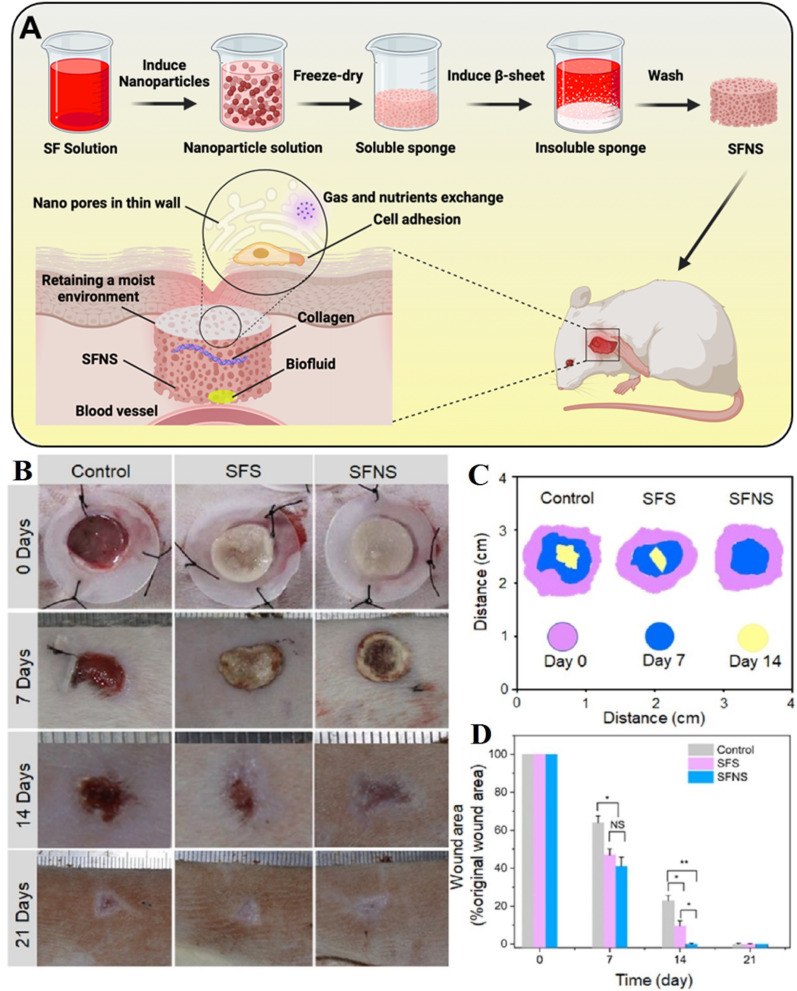
NPMs in wound healing: (A) a schematic showing SF sponges with nanoporous walls (SFNS), derived from SF nanoparticles, which facilitate wound repair through multiple mechanisms. Modified from ref. 159. Created with Biorender.com; (B) the progression of wound healing over 21 days in a rat skin wound model, comparing SF sponges without nanopores (SFS) and SF sponges with walls containing nanopores (SFNS), demonstrating superior healing with SFNS; (C) progression of wound bed closure over a 14-day period across different treatments; (D) determination of wound areas using ImageJ (*p < 0.05, **p < 0.01). This figure has been adapted/reproduced from ref. 159 with permission from the American Chemical Society, Copyright 2023.

The wound-healing process is a natural biological activity in the human body that includes four overlapping phases: hemostasis, inflammation, proliferation, and tissue remodeling or resolution.^157^ Wound healing typically occurs through a series of phases with specific physiological functions, following a specific sequence, timing, and duration at an optimal intensity.^158^ Developing novel nanoporous structures for wound healing has attracted considerable attention in recent years to enhance sterility and process acceleration. Fig. 7A illustrates a schematic of SF sponges with nanoporous walls, derived from SF nanoparticles, that facilitate wound repair through multiple mechanisms. Fig. 7B shows the progression of wound healing over 21 days, demonstrating significant improvements in the wound area. Fig. 7C demonstrates the progression of wound-bed closure over a 14-day period for each treatment group, and Fig. 7D shows wound areas, highlighting improved healing results.^159^

Managing bleeding alongside sterilization is also of concern in terms of wound healing. Excessive blood loss leads to hemorrhagic shock if it is not controlled immediately.^160,161^ Controlling bleeding caused by battles, surgeries, and accidents is significant as it might lead to less mortality.^162^ Regarding this issue, nanoporous fiber-reinforced composite scaffolds (NFRCS) have been developed. These scaffolds utilize a hemostatic film-forming composition (HFFC) as a continuous phase and a randomly dispersed cotton gauze (Ct) phase, enabling versatile formats such as a bandage or roll form and a wristband. *In vivo* experiments have shown that increased coagulation results from enhanced Cp NFRCS, which entails Cp (CHT with PVA) HFFC. Moreover, according to its nanoporous structure, it can act at the cellular level, giving rise to improved wound healing.^163^

**Fig. 8:**
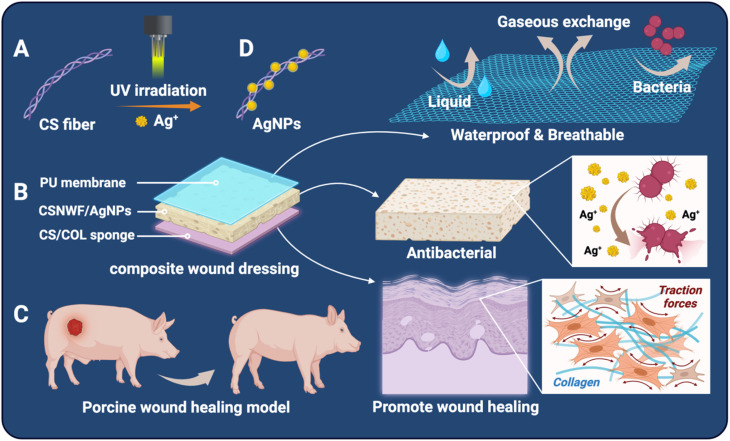
Schematic representation of the AgNP-anchored sandwich structure composite wound dressing (PU-CSNWF/AgNPs-CS/COL). (A) *In situ* synthesis of AgNPs anchored onto the CSNWF fibers. (B and C) The schematic picture of a composite wound dressing shows its sandwich structure and purpose of application. (D) A healed porcine model suffering from burn injury. Modified from ref. 165. Created with Biorender.com.

Apart from significant results of monitoring wound healing in common animal wound models, such as rats and rabbits, it should be highlighted that selecting a model that has the most concordance of the wound healing process with humans is crucial. According to some studies, human and small mammal cutaneous tissues are only 53% similar in wound healing mechanism, whereas this concordance ascends to 78% in humans *versus* pigs.^164^ In a study by Yang and colleagues, the effectiveness of applying an *in situ*-synthesized composite wound dressing on a porcine model with severe burn injury was confirmed. Regarding that, the composite wound dressing was designed with a sandwich structure comprising three layers (Fig. 8).^165^ The outer layer was a nanoporous polyurethane (PU) membrane, providing high stretchability and acting as a barrier against external contamination (bacteria, oil, water, and dust) while remaining permeable to water vapor. The inner layer is a sponge prepared with CS and collagen (COL), leading to great biocompatibility and biodegradability according to the coagulation of CS and cytocompatibility of COL. The porous structure of the CS/COL sponge removes secretions and keeps the wound environment moist. The interlayer is a silver nanoparticle (AgNP) anchored CS nonwoven fabric (CSNWF), which provides a sustained release of Ag^+^ with no direct contact with wound tissue, thereby reducing the toxicity of the AgNPs. This structure has shown great antibacterial activity against *E. coli*, *Pseudomonas aeruginosa*, and *S. aureus*. In addition, it was shown that the efficient expression of VEGF and endothelin, along with inhibition of NO production, could promote angiogenesis.^165^

In recent years, 3D carbon nanostructures have received much more attention among nanocarbons due to their flexible characteristics and intrinsic potential, including chemical inertness, low density, versatile porosity, high internal surface area, lightweight, low thermal conductivity, and electrical conductivity.^166–169^ In a study by Shukla and coworkers, a graphene aerogel (GA) with a nanoporous honeycomb-like structure was synthesized from crude biomass. This graphene framework has shown wound healing ability as a cell growth promoter on human lung bronchial epithelial cells, HaCaT, and COS-7. Concerning that, cell viability assays, including MTT and lactate dehydrogenase (LDH) assays, were performed. Cell viability was not affected significantly at different doses of GA ranging from 1 to 20 mg mL^−1^. Moreover, the wound healing ability of GA was observed through an *in vitro* experiment on the scratched epithelial cell surfaces by increasing cell migration at the lowest dose (1 mg mL^−1^). The results indicated that different doses of GA did not affect cell viability, and the higher dose (10 mg mL^−1^) efficiently promoted wound healing within 12 h by stimulating cell growth in the scratched area. Western blot analysis confirmed a dose-dependent increase in β-catenin and E-cadherin expression.^170^ These proteins play a crucial role in wound repairing, proliferation, alveolar differentiation, development, morphogenesis, and alveolarization of epithelial cells confirmed by immunoblotting results.^171^ Other structures that have shown promising results in terms of wound dressing materials are hydrogels. Some of the sophisticated functions of hydrogel dressings offer features such as antimicrobial activity, adhesion, hemostasis, anti-inflammation, antioxidant properties, drug delivery, self-healing capabilities, and conductivity.^172^

The hydrogel films included sodium carboxymethylcellulose (NaCMC) and hydroxypropylmethylcellulose (HPMC) crosslinked with citric acid through ester bonds. This interconnected nanoporous network has demonstrated promising results, with remarkably higher drug loading efficiency for cationic drugs (methylene blue) compared to anionic drugs (tetracycline). The hydrogel films provided sustained drug release over 72 hours. Furthermore, antibacterial activity was observed within three days at 37 °C in PBS, indicating that these hydrogels are promising candidates for wound-healing drug delivery systems.^173^ All in all, wound healing is considered a significant process that should effectively occur in each body. Meanwhile, different nanoporous structures have shown excellent potential in different medical applications. Therefore, novel practical nanoporous constructions could be designed and subsequently investigated through particular experiments to improve different phases of wound healing, which plays a substantial role in every individual's health.

### 4.3. Drug delivery

In recent years, the application of nanocomposites as drug delivery vehicles has seen significant growth in both academia and industry.^174^ These nanostructured materials often exhibit superior properties compared to traditional solid matrix materials or micro/macro-composites. Among these prominent features are mechanical features, controlled drug release profiles, and desirable engineering characteristics (*e.g.*, thermal stability, chemical resistance, and optical clarity). Sustained drug release maximizes therapeutic efficacy and improves patient satisfaction.^175^

The development of modified drug delivery (MDD) to reduce the bioavailability and side effects of other DDSs, as well as to control drug release and focus more on its release site, has achieved significant success so that one of these platforms, called ordered nanoporous carbon (ONC) structures, has been considered as a new generation of drug delivery and biomedical systems. The structure of ONC, having a large number of regular pores, has led to better control of drug release and increased drug delivery stability, maintaining a tremendous amount of nutrients. Considering the importance of this nanostructure, cephalexin (CFX) was loaded into ONC functionalized with amine groups (ONC-A) and ONC; thus, CFX-ONC-A and CFX-ONC nanocomposites were prepared. Based on the results, the biocompatibility of both nanocomposites against human colorectal adenocarcinoma (Caco-2) cells has been reported to be more than 80%. The release mechanisms of these nanocomposites depended on pH, with total releases at gastric pH = 1.2 of 91% for ONC-F and 85% for ONC-AF, and at intestinal pH = 6.8 of 85% for ONC-F and 58% for ONC-AF, achieved over 12 hours. In contrast, pure CFX released 100% at pH = 1.2 in 0.5 h and 97% at pH = 6.8 in 1 h. Both systems exhibited a rapid initial burst release in the first hour, followed by a slower sustained release over the subsequent 11 hours, governed by Fickian diffusion as described by the Weibull model. This controlled release is attributed to the ordered nanoporous structure of the carbons, which allows drug molecules lodged within the mesopore channels to diffuse gradually toward the dissolution medium.^6^

Pulmonary drug delivery and improving its performance by using porous particles and nanosystems to increase bioavailability in respiratory diseases and lung cancer have always been fundamental goals of biomedicine. For this purpose, Zhou *et al.* used MOFs, a novel class of porous materials featuring pores typically smaller than 2 nm, arising from the periodic network structure of metal–organic coordination bonds. The main focus has been on a type of MOF called cyclodextrin-based MOF (CD-MOF), consisting of potassium ions and γ-CD. They loaded ketoprofen (KTP), an acidic drug with pH-dependent solubility, into CD-MOF with a pore width of 1.3 nm and studied the excellent biocompatibility of the KTP-CD-MOF nanocomposite through *in vitro* cytotoxicity assays on human lung cell lines (A549 and Calu-3) and *in vivo* evaluations in SD rats. In 2 hours, the release rate of ketoprofen was 100%, while the amount of ketoprofen released from the nanocomposite was 89%, and the cell viability values were approximately 100% in all concentration ranges for CD-MOF alone. Furthermore, the biocompatibility of CD-MOF was rigorously assessed through *in vivo* inhalation toxicity studies in rats, measuring inflammatory cytokines in bronchoalveolar lavage fluid (BALF). The absence of significant increases in the levels of MIP-1α, IL-6, IL-1β, and TNF-α compared to the blank control (with only mild elevations noted for the commercial carrier at 48 h) demonstrated the excellent biocompatibility of this composite, attributed to its homogenous nanoporous structure that minimizes irritation. Histological analysis further confirmed no obvious inflammation or tissue damage in lungs or major organs (heart, liver, spleen, and kidneys) post-administration, reinforcing CD-MOF's safety profile. *Ex vivo* fluorescence imaging using rhodamine B-loaded CD-MOF revealed high lung deposition rates, with gradual metabolism *via* liver and kidney, highlighting its potential as a versatile carrier for sustained pulmonary drug delivery.^176^

The integration of nanosystems and nanoparticles into ocular drug delivery systems has always been necessary, considering the significant advances in this field and the influential role of nanosystems and nanoparticles in various DDSs. Moreover, as mentioned in the previous example, CD-MOFs have significantly contributed to the formation of effective nanocomposites in DDSs by creating a suitable substrate of porous space and the possibility of loading drugs with specific weight and volume, so a 2D nanosheet (NS) of γ-CD-MOF was synthesized and loaded with dexamethasone (DXM), resulting in the formation of DXM-NS-MOF and DXM-CD-MOF nanocomposites. The results showed that the release pattern in rabbit aqueous humor cells, with a temperature-dependent release mechanism, occurred in fast and slow modes. In the first 24 hours, it is 73% and 55% for DXM-CL-NS-MOF and DXM-CL-CD-MOF nanocomposites, respectively, which could be attributed to their cubic structure and increased contact of the drug with the environment. After four days, DXM is wholly removed from the MOF porous cavity in a slow state. Finally, by examining the effect of the formulation of eye drops made with these nanocomposites on the structure of the cornea and the improvement of inflammation, it was found that after seven days of use, the primary inflammation was resolved. During this time, no irritation was observed in the cornea and eye surface tissue.^177^

Along with radiation therapy, hyperthermia or its combination could improve the effectiveness of chemotherapy. On the other hand, chemotherapies based on old methods have always been associated with the risk of damage to healthy cells, so to achieve these two goals, DDSs using nanoparticles (NPs) have been investigated, and Fe_3_O_4_ NPs were one of the structures used in hyperthermia due to their suitable magnetic properties. For this reason, Khan and Kumar Sahu synthesized PEG-dicarboxylic acid (PEG-D) coated aqueous dispersible mesoporous magnetic nanoclusters (MNCs). Examination of the morphologies of MNCs has shown that they possess a highly porous structure, one of the main characteristics of nanocarriers in DDSs. They have shown better thermal performance at higher concentrations, depending on the existing magnetic field. Thus, DOX was loaded into amidated MNCs. In the DOX-Fe_3_O_4_-MNC nanocomposite, the mechanism of release and function occurs in two ways in 24 h, and this mechanism is initially dependent on pH, and the NPs slowly dissolve in the acidic environment around the tumor by releasing Fe ions and forming ROS; on the other hand, the magnetic field increased the temperature from 41 to 43 °C, which caused the release of DOX from the porous space inside the NP. Based on the results, the toxicity effect of this nanocomposite on breast cancer (MCF-7) cells was 60% at a concentration of 1000 µg mL^−1^. Therefore, the MNCs could be a suitable alternative to treat cancer cells.^178^Table 3 shows types of nanoporous material-based composites for drug delivery and release mechanisms.

**Table 3:** Types of nanoporous material-based composites for drug delivery and release mechanism^a^

Nanocomposite	Porous part	Synthesis method	Critical analysis	Pore size	Drug	Target	Mechanism of targeting and release	Significant outcome	Ref.
Poly(ethyleneglycol)-bis-(carboxymethyl)-ether (PEGD)-functionalized Fe_3_O_4_ mesoporous magnetic nanoclusters (MNCs)	MNCs	Solvothermal process, followed by magnetic separation	XRD, FTIR, Raman spectroscopy, VSM, FESEM, TGA, EDX, zeta potential analysis, BET, and BJH techniques	3.4 nm	DOX	MCF-7	pH-responsive	High DOX encapsulation efficiency of ∼92%; effective conjugation of DOX onto the MNCs and their potential for generating hyperthermia	178
Micelle-entrapped nanoporous silica xerogel (ITZ-M40-silica)	Silica xerogel	Biomimetic synthesis using micelles as templates for silica xerogel formation	SEM, FTIR, DSC, nitrogen adsorption/desorption, UV-vis spectrophotometry, optimized experimental design software (Design Expert), and AlamarBlue assay	2–5 nm	ITZ	Caco-2 cell (model cells for intestinal absorption)	Primarily diffusion-controlled	Superior drug dissolution and improved oral absorption; safe for Caco-2 cells	179
				5–10 nm					
				10–50 nm					
Zirconium MOF (5-FU-MOF-801)	MOF	*In situ* encapsulation approach; reaction of ZrCl_4_ and fumaric acid	FTIR, PXRD, FE-SEM, EDAX, UV-vis spectroscopy, NMR, TGA, HPLC, BET, Monte Carlo simulation, RDFs, configuration snapshot analysis, MTT assay, fluorescence imaging/flow cytometry	Tetrahedral cavities of 4.8 and 5.6 Å, and an octahedral cavity of 7.4 Å	5-FU	Human colon cancer cell (SW480) and human embryonic kidney cells (HEK-293)	pH-dependent release at pH 5.0 and 7.4, fitting the Hixson–Crowell model	High 5-FU loading capacity and pH-dependent drug release behavior; enhanced therapeutic efficiency against cancer cells	180
Poly(l-lactide-*co*-ε-caprolactone) (S-PLCL) membrane	Fibrous membranes	Electrospinning	SEM, water contact angle and water vapor permeability assessments, UV-vis spectroscopy, antimicrobial activity (colony-forming units), NMR, DSC, GC-FID, ICP-OES, and Karl Fischer titration	N. A.	TC	HOF and HOK	Fickian diffusion mechanism	Effective antibacterial activity against oral plaque samples, maintaining high drug concentrations over time	181
Zn-based MOFs: CCM-DMOF-1 and CCM-DMOF-1-NO_2_	MOF	Reflux method and impregnation method for drug encapsulation	FT-IR, PXRD, NMR, N_2_ adsorption (BET), SEM, UV-vis spectroscopy, fluorescence microscopy, and computational simulation (BIOVIA Material Studio)	1.7–1.9 nm	CCM	Gastric cancer cells (AGS) and HEK-293 cells (healthy model)	Hydrolysis (phase transition from a 3D to a 2D soluble structure, facilitating drug release)	Improved the solubility of CCM and enhanced cytotoxicity against cancer cells (AGS) in comparison with free CCM	182
Cu-BTC MOF (CHX–Cu-BTC)	Cu-BTC MOF	Cu-BTC was synthesized by dissolving Cu(NO_3_)_2_·3H_2_O in water and H_3_BTC in DMF; CHX was loaded onto Cu-BTC by a simple impregnation method	FT-IR, XRD, BET/BJH, TGA, SEM, EDX, TEM, zeta potential	N. A.	Chlorhexidine (CHX)	*E. coli* and *S. aureus*	Release triggered by ionic strength, pH, and diffusion through pores (electrostatic adsorption)	Synergistic antibacterial effect of CHX-Cu-BTC against Gram-positive and Gram-negative bacteria	183
NPSNPs loaded with DMOG	Silica nanoparticles	A modified synthesis method, including centrifugation, washing, drying, and calcination; DMO was loaded onto NPSNPs *via* physical adsorption	TEM, BET, TGA, zeta potential, UV-vis spectroscopy, ELISA, ALP assay, resazurin-based cell viability assay, and endothelial tube formation	3 nm	DMOG	ASCs, HUVECs	Desorption/electrostatic interaction-based release	The system is a suitable tool for achieving sustained chemically induced hypoxia. The sustained DMOG release led to a significantly higher and more constant VEGF secretion rate from ASC compared to free DMOG, promoting vascularization	184
COF encapsulated with TMP (TMP–COF)	COF	Two-step self-assembly synthetic method, followed by stirring the COF in the drug solution	FESEM, FTIR, XRD, BET/BJH, UV-vis spectroscopy, zeta potential, MIC test, antibacterial assay	2.04 nm	TMP	*S. aureus* and *E. coli*	Diffusion-controlled (primarily governed by the Higuchi model)	Demonstrated high antibacterial efficiency; the release percentage reached approximately 95% after 4 days	185
Zr-based MOF (UiO-66-NH_2_) modified with poly(amidoamine) (PAMAM-G3) dendrimer and functionalized with d-galactose (DOX-CUR-Gal-PAMAM-UiO-66-NH_2_)	Zr-MOF structure	Solvothermal method for UiO-66-NH_2_; Michael addition and amidation reactions for PAMAM dendrimer growth; and imine condensation reaction for functionalization	DLS/zeta, FTIR, SEM, EDX, TEM, AFM, UV-vis and PL spectroscopy, XRD, BET/BJH, MTT assay (cell viability), DAPI staining, and cell cycle analysis	5.87 nm	DOX and CUR	Hepatic cancer cells (HepG2) and HUVECs	pH-dependent release; d-Gal functionalization targets ASGPRs	Achieved high co-loading capacity (DOX: 71.4%, CUR: 56.0%); suitable for bioimaging and targeted tumor therapy	186
OMCs, either nitrogen-doped (C_AP_ samples) or functionalized with CS (C_CS_ samples)	OMCs	Soft-templating method and hard-templating method, followed by carbonization, template removal, oxidation, and subsequent CS grafting	XRD, TEM, SEM, elemental analysis, BET/BJH, Boehm method, FTIR, PAMPA (permeability), UV-vis spectrophotometry	1.67– 9.22 nm	Losartan potassium (LOS)	N. A.	pH responsiveness (electrostatic repulsion)	CAP samples showed rapid release. CCH samples exhibited sustained/controlled release, resulting from drug affinity to the polymer and pore blockage	187
Hybrid nanoporous anodic alumina gradient-index filters (Hy-NAA-GIFs)	NAA	Heterogeneous anodization process combining sinusoidal current-density anodization and constant potential anodization	FESEM, reflectance spectroscopy, UV-vis spectrophotometry	39 ± 3 nm	DOX	None specified (analyzing kinetics *in vitro* under dynamic flow conditions)	Diffusion-controlled	Successfully allowed real-time monitoring and detailed analysis of DOX release kinetics; increasing the pore length resulted in an increase in the diffusion time constant	188
Bi-functionalized aminoguanidine-PEGylated PMO nanoparticles (RNP–AGu–PEG 1500-PMO)	PMO	M-PMO was prepared *via* a hydrothermal method, followed by acidic extraction to remove the template; surface modification included covalent grafting of PEG and subsequent functionalization with AGu moieties	TEM, SEM, BET/BJH, TGA, XRD, ATR-FTIR, DLS/zeta potential, MTT assay, and CLSM	9.3–11 nm	Cas9-sgRNA ribonucleoprotein complex (RNP)	Human fibrosarcoma cells (HT1080-GFP)	The proton sponge effect of aminoguanidine, which facilitates endosomal escape	Achieved efficient intracellular delivery and a high gene-editing efficiency of about 40% (GFP gene knockdown); the large pore size and tailored surface chemistry make PMOs suitable for high loading of proteins like RNP	189
Biogenic Mg-calcite from blue crab shells	Biogenic Mg-calcite (mesoporous material)	Ball-milling of crab shells into powder, mixing with 5-FU solution, and compressing into tablets	Confocal micro-Raman spectroscopy (CRM), XRD, SEM, BET, surface-enhanced Raman scattering (SERS)	3–60 nm	5-FU	None specified; intended for eventual targeted colon delivery (lower GI tract)	Diffusion-controlled	Demonstrated slow release of 5-FU. Confirmed that the drug was incorporated inside the nanopores, as no drug signal was detected on the tablet surface by Raman or XRD	190
GIC containing nanoporous silica (GIC-NPS)	NPS	Mixing NPS into the powder component of GIC before mixing with the liquid component and molding	Evaluation of cationic ion capture and dye release (UV-vis spectroscopy), rechargeability, and mechanical properties	2–4 nm	Rhodamine B (RhB), a cationic water-soluble dye	Not explicitly mentioned (intended for dental applications to prevent caries)	Sustained release of positively charged RhB attracted to the negatively charged surface of silica	The amount of dye released from the complexes increased with decreasing pore size. The mechanical property (compressive strength) of GIC was slightly improved by the addition of NPS compared to the control GIC	191
Nanoporous optical antenna (NOA) composed of an inorganic nanoporous silica vehicle integrated with gold nanorods (AuNRs)	Silica vehicles	Standard synthetic procedure (involving CTAB, EG, TEOS, and APS) followed by APTES functionalization and deposition of an Au layer using E-beam evaporation	UV-vis spectroscopy, MTT assay	N. A.	AMC	HeLa cells and MCF-7 cells	Photo-responsive	Provided an advanced engineering strategy for forcing the release of payloads, thereby overcoming the physical intracellular barriers that lead to resistance	192
CS/polyvinylpyrrolidone/γ-alumina/quercetin (CS/PVP/γ-alumina/QC)	γ-Alumina	Sol–gel method for γ-alumina; chemical cross-link with glyoxal; double oil in water (W/O/W) emulsification method for core–shell	XRD, FTIR, DLS, FE-SEM, zeta potential measurements, and UV-vis spectrophotometry	N. A.	QC	MCF-7 cancer cells	pH-sensitive (swells in acidic environments (like tumour microenvironments, pH 5.4))	Improved encapsulation efficiency up to 95%; significant cytotoxicity (9% cell viability) and high apoptotic cell death (95.33%)	193
Monodispersed nanoporous amorphous titania submicron particles with europium(iii) acetylacetonate (Eu(acac)_3_)	Amorphous titania	Microfluidic technique to create well-defined titania-ODA hybrid submicron particles, followed by solvent extraction	N_2_ adsorption–desorption isotherm (BET), FTIR, TEM, FE-SEM, EDX, TEM, XRF, XRD, photoluminescence spectroscopy and fluorescence microscopy, UV-vis spectroscopy	1–1.2 nm	IBU	N. A.	Diffusion of solvent molecules into titania pores	Controlled release of IBU, with most released within 6 hours, and slower release with PEG-silane modification, as a bioinert DDS carrier	194
HAP and GG	HAP	Simple chemical precipitation method; sintering at 600 °C	XRD, FTIR, XPS, TGA, FESEM, TEM, NMR, N_2_ adsorption–desorption isotherm (BET)	33 Å	Naringenin	*S. aureus*, *E. coli*, McCoy (normal bone cells) and Saos-2 (osteosarcoma cancer cells)	Diffusion from the porous HAP matrix	Stable and controlled release of naringenin; effective antibacterial activity; significant inhibitory activity against osteosarcoma cells	195
PLGA capsules containing light-activated liposomes and AuNRs	Nanoporous PLGA capsules	Modified reverse-phase evaporation (REV) method, followed by attachment of AuNRs and lyophilization	TEM, SEM, UV-vis spectrometry and *in vivo* fluorescence imaging	Less than ∼2 nm	Methotrexate (MTX) and Cy5 fluorescent dye	For PVR and noninfectious uveitis (New Zealand white rabbits)	Pulsed near-infrared (NIR) laser	Development of a dosage-controllable drug delivery system that is safe for the retina and can provide effective and dose-controllable drug release *in vitro* and *in vivo*	196
Folate-conjugated hollow polymeric capsules (FA-HPCs)	HPCs	A one-pot Friedel–Crafts reaction, followed by conversion of hydroxyl groups to carboxyl groups and conjugation with FA using the EDC-NHS method	SEM, TEM, EDAX, BET, FTIR, TGA, UV-vis spectrophotometry, and confocal laser scanning microscopy (CLSM)	3–5 nm	DOX	MCF-7 cells and mouse embryonic fibroblast cells (NIH/3T3)	pH-Responsive	A high drug encapsulation efficiency of up to 86% and controlled drug release	197
ZIF-8 (MOF)/poly(γ-glutamic acid) hybrid nanocomposite (ZIF/PGA-C/M)	ZIF-8	Polymerization and hydrolysis of PGA, followed by crosslinking under a nitrogen atmosphere	^1^H NMR, FT-IR, XRD, XPS, DLS, TEM, SEM, TGA, and DPBF degradation assay	Below 10 nm	Ciprofloxacin (CIP) and methylene blue (MB)	Methicillin-resistant *Staphylococcus aureus* (MRSA) and HUVEC	pH/enzyme-responsive (lipase)	The synergistic efficacy of the nanocomposite against MRSA infection in a mice skin infection model, resulting in quicker wound healing	198
Zr-based MOF UiO-66, functionalized with designed N_3_-PEG-PO_3_ ligands	UiO-66	Solvothermal reaction, followed by surface functionalization of UiO-66 with N_3_-PEG-PO_3_ ligands	SEM, TEM, DLS, FTIR, TGA, NMR, UV-vis spectroscopy, PXRD, HPLC-MS, and flow cytometry	11 Å (octahedral) and 8 Å (tetrahedral)	DOX	HeLa, MDA-MB-231, and MCF7	Enzyme-responsive (based on the enzymatic activity of ALP)	Enhanced drug release was observed in cells with higher ALP expression	199
Dually cross-linked hydrogel consisting of a self-assembling peptide (IDM-1) and chemically cross-linked CS with genipin	Hydrogel network	Solid phase peptide synthesis (SPPS) techniques, peptide self-assembly and chemical cross-linking for the CS framework	HPLC, MS, NMR, circular dichroism (CD) spectroscopy, rheological studies, TEM, SEM, and histological analysis	N. A.	Indomethacin	RAW 264.7 (macrophages), NIH3T3, and rat blood	Enzyme-responsive (protease-triggered degradation responsive to the wound microenvironment)	Exceptional mechanical properties, hemostatic control, antibacterial efficacy, and accelerated wound healing	200
PUU with hydroxyapatite nanoparticles (nHAp)	Hydroxyapatite NPs (nHAp)	ROP and reaction with 1,4-diisocyanatobutane (BDI) and GSSG-OMe4 to form the PUUs	SEC, ^1^H-NMR, FT-IR, DSC, SEM, EDX, and a colorimetric assay (NTSB)	N. A.	DOX	Mouse embryo osteoblast precursor cells (MC3T3-E1) and HCT-116 cancer cells	Redox-responsive and pH-responsive	A sustained and redox-responsive release of Dox, with less than 30% of the drug released over 21 days, with a high cytotoxic effect on cancer cells	201
Organosilica NPs (HMONs) and GOQDs	HMONs–GOQD	Sol–gel method and the covalent coating of HMONs with GOQDs	TEM, SEM, TGA, DLS, UV-vis absorption analysis (BET)	3.94 nm	DOX	Breast cancer cell line (4T1)	Photo-responsive	Enhanced local drug release due to photo-responsive degradation	202
MS-coated carbon nanotubes (CNTs)	MS shell	Coating CNTs with a MS shell using a sol–gel procedure, modifying with isobutyramide grafts, and wrapping with a human serum albumin	TEM, nitrogen adsorption–desorption isotherms and zeta potential measurements	3.4 nm	DOX	Murine mammary carcinoma (D2A1)	NIR light-responsiveness (photothermal effect)	Combined phototherapy and NIR-light-mediated drug release of DOX, with a high drug loading capacity	203
CS and poly(*N*-isopropylacrylamide), gold and magnetic (Fe_3_O_4_) NPs	Nanogel hydrogel NPs	*In situ* preparation of AuNPs on CS using a green hydrothermal method, followed by radical polymerization	FTIR, EDAX, XRD, DLS, SEM, HRTEM	N. A.	Azithromycin & DOX	MCF-7 and A431	Visible light-sensitive (AuNPs act as photothermal transducers)	Demonstrated efficient drug release (up to 55% for optimized samples) triggered by green light	204
MIL-88B-NH_2_ with Fe_3_O_4_ magnetic NPs	MIL-88B-NH_2_ (MOF)	Optimized approach, using acetic acid as a modulating agent and F127 co-polymer as a stabilizing agent in an aqueous medium	SEM, TEM, PXRD, zeta potential, BET, VSM, confocal laser scanning microscopy (CLSM)	16.9 nm	Carmustine (BCNU) and mertansine (DM1)	Luciferase expressing U251 glioblastoma cells	AMF-triggered controlled release	Development of a nanocomposite with superparamagnetic properties, demonstrating on-demand controlled drug release triggered by AMF	205
GO, Fe_3_O_4_, PNIPAM, AAm, and alginate	Nanocomposite hydrogel (NCH)	Inverse chemical co-precipitation method for Fe_3_O_4_ on GO, and centrifugal microfluidic method, followed by NIPAM polymerization	TEM, DLS, XRD, VSM, XPS, DSC, and live/dead fluorescence staining	N. A.	DOX	MCF-7 cells	NIR light-, magneto-, and pH-responsive	Sustained and pH-triggered DOX release; increased DOX release by NIR light and AMF application; effective killing of cancer cells	206
Zinc ferrite-mesoporous zinc oxide core–shell (ZnFe_2_O_4_–mZnO) nanostructures decorated with graphitic (g) carbon nitride (Ox-p-g-C_3_N_4_) nanosheets	mZnO-ZnFe_2_O_4_-p-g-C_3_N_4_	Calcination/chemical oxidation procedure of melamine, followed by ultrasonic exfoliation; sol–gel route	FT-IR; XRD; FESEM; EDX; TEM; DLS and zeta potential analysis; VSM; N_2_ adsorption/desorption (BET)	Mesopores with sizes of 5–100 nm	CUR	Primary human fibroblast and A549 lung carcinoma cell line	Dual pH- and ultrasound-responsive release	Enhanced targeted endocytosis with the charge-reversal ability for anticancer drug delivery	207
Chondroitin sulfate networks with encapsulated polypyrrole (PPy) colloids	PPy NPs	Self-crosslinking of chondroitin sulfate *via N*-ethyl-N 0-(3-dimethylaminopropyl) carbodiimide chemistry in the presence of PPy colloids	FT-IR, SEM, swelling kinetics and drug release measurements	N. A.	5-FU	Mouse fibroblast cell line (L-929) and human cervical cancer cells (HeLa)	Electrical responsiveness	Electro-responsive ON/OFF 5-FU release pattern, achieving a maximum cumulative release of up to 90% with applied pulse potentials	208
Methacrylated hyaluronic acid (HA/MA), rGO, and PANI	rGO NPs	*In situ* polymerization for HA/MA-rGO, post-polymerization for PANI	FT-IR, NMR, Raman spectroscopy, SEM, DLS, zeta potential analysis, and compressive strength tests	N. A.	IBU	HaCaT cells	Electro-stimulated	Enhanced electrical conductivity and mechanical performance, allowing for electro-responsive drug release	209
Magnetic Fe_3_O_4_/polyaniline (PANI) composite particles	Fe_3_O_4_ core and particle surface structure	A three-step process; solvothermal process, followed by modification with glycine, and then chemical oxidative polymerization of aniline	SEM, TEM, BET, XRD, XPS, TGA impedance analysis, magnetic susceptibility measurements	3.79 nm	Trypsin (TR), albumin (AL), and *p*-acetamidophenol (*p*AP)	*E. coli*, *S. dysentery*, *S. sonniia*, *S. brodie*, *B. cereus*, and *K. pneumonia*	Electric current responsiveness	Strong paramagnetic (47.77 emu g^−1^) and semi-conducting properties, and the release of up to 33–88% of adsorbed biomolecules per drug	210
CS rhIFNα-2b carboxymethyl nanoporous microspheres (CS-rhIFNα-2b-CCPM)	CS-rhIFNα-2b-CCPM	Column method, with ethyl acetate as a pore-forming agent, and electrostatic self-assembly technique	SEM, SDS-PAGE, CD, Bradford method, and ELISA method	300–400 nm	rhIFNα-2b	Human lung adenocarcinoma cell line (A549)	Ion-responsive *via* ion exchange	Sustained release of rhIFNα-2b; prolonged the half-life of rhIFNα-2b *in vivo*; obvious lung-targeting effect	211

a Abbreviations: AAm, acrylamide; ASCs, adipose tissue-derived stem cells; ALP, alkaline phosphatase; AMF, alternating magnetic field; AFM, atomic force microscopy; BJH, Barrett–Joyner–Halenda; BET, Brunauer–Emmett–Teller; CFP, capillary flow porometry; CS, chitosan; CD, circular dichrogram; CUR, curcumin; DXMT, dexamethasone; DSC, differential scanning calorimetry; DMOG, dimethyloxalylglycine; EDS, energy dispersive spectroscopy; FESEM, field emission scanning electron microscopy; FTIR, Fourier-transform infrared; GE, gelatin; GOQDs, graphene oxide quantum dots; GG, gum ghatti; HAEC, human aortic endothelial cells; HDMVE, human dermal microvascular endothelial cells; HUVECs, human umbilical vein endothelial cells; HAP, hydroxyapatite; MS, mesoporous silica; NAA, nanoporous anodic alumina; NPSNPs, nanoporous silica nanoparticles; NIR, near infra-red; om-CMS, ordered mesoporous calcium magnesium silicate; PANI, polyaniline; PCL, polycaprolactone; PEG, polyethylene glycol; PEO, polyethylene oxide; PLA, polylactic acid; PLGA, poly(lactide-*co*-glycolic acid); PNIPAM, poly(*N*-isopropylacrylamide); PPy, polypyrrole; PUU, polyurethane-urea; QC, quercetin; rMSCs, rat mesenchymal stem cells; ROP, ring opening polymerization; SEM, scanning electron microscopy; SCs, Schwann cells; SF, silk fibroin; TIPS, thermal inversion phase separation; TGA, thermogravimetric analysis; TEM, transmission electron microscopy; TMP, trimethoprim; VEGF, vascular endothelial growth factor; VSM, vibrating sample magnetometer; WCA, water contact angle; XRD, X-ray diffraction.

With the passage of time and the advancement of science in the field of modern drug delivery, researchers have achieved very useful results. Normally, a large dose of the drug enters the body at the very beginning of use and decreases after a few hours, which leads to repeated use. DDSs based on nanocomposites have solved this problem because, in this model, they release a fixed amount of drug in a certain place and control its release, which is very important in the process of treatments such as chemotherapy. Also, one of the advantages of these nanocomposites is to protect the drug molecule and increase the efficiency of drug therapy, which ultimately led to their popularity.

## 5. Future directions, challenges, and concluding remarks

NPMs stand as silent revolutionaries, pivotal innovations, and promising classes of materials for various biomedical applications. Their intricate structures, high surface area, tunable porosity, and biocompatibility have unlocked new frontiers in diagnostics, drug delivery, and tissue regeneration. Despite the potential of NPMs, several challenges remain. As we delve deeper into their promise and address challenges, we envision a future where these nanostructures wield outsized influence, transforming healthcare and improving human lives. The journey continues, fueled by innovation and the unwavering pursuit of better health outcomes, enabling breakthroughs in tackling major health challenges.

The synthesis of these materials frequently includes intricate procedures that may result in batch-to-batch variability, making reproducibility a concern. Investigation of new materials with biocompatibility, biodegradability, and properties will be improved using customized design and eco-friendly synthesis methods. The synthesis of NPMs typically requires intricate procedures that may prove challenging to expand for mass production without compromising uniformity. Development of effective ways that maintain their desired properties presents a major challenge for bringing them to market.

It is still a significant challenge to make sure that NPMs do not cause adverse responses in biological systems. The long-term safety and biocompatibility of these materials in the body are critical. Moreover, it is important to consider the durability of nanoporous structures in biological settings to maintain effectiveness over time. Conducting long-term *in vivo* studies to better understand the biodistribution, metabolism, and safety of NPMs will be essential for their clinical translation. Further *in vivo* investigation is necessary to fully elucidate the biodistribution, metabolism, and safety of NPMs to facilitate their clinical transition. Accomplishing accurate modification of nanoporous surfaces to improve targeted interactions with biosystems at the cellular and molecular levels is still a technical obstacle in predicting their behavior *in vivo*.

It is also crucial to improve the biocompatibility, efficacy, and understanding of how NPMs interact with biological systems by developing new functionalization strategies, as well as ensuring their safe degradation or elimination. Manufacturing NPMs in bulk while maintaining their quality and stability remains difficult. Developing cost-effective production methods is crucial for their widespread adoption. It is essential to guarantee the physicochemical stability of NPMs under physiological conditions to ensure their dependable use.

The regulatory landscape for new materials is intricate and can impede the translation from lab studies to practical applications in healthcare. Consistently and effectively functionalizing NPMs to meet specific biomedical requirements can be technically demanding.

Integrating NPMs with biosensors or bioimaging tools to create multifunctional platforms can revolutionize treatment methods by combining multiple modalities. Furthermore, the integration of 3D printing, microfluidics, and artificial intelligence with nanoporous structures can enable new possibilities in the fields of diagnostics and therapeutics.

Future studies could extensively focus on engineering NPMs that are responsive to exogenous and endogenous triggers (*e.g.*, pH and temperature) to enhance the efficiency of delivering drugs to specific targets. Indeed, their ability to react to physiological changes can lead to dynamic drug release systems. Incorporating NPMs into advanced biomedical devices, like sensors and implants, has the potential to transform how diseases are monitored and treated. Highlighting green chemistry and sustainable production techniques will be crucial for minimizing the environmental impact of developing these materials.

By tackling present obstacles and investigating new pathways, the realm of NPMs has the potential to make a substantial impact on the progress of biomedical science, ultimately improving healthcare solutions and patient care. In summary, although NPMs offer numerous possibilities in the biomedical field, it will be crucial to tackle the related hurdles and investigate future paths to fully utilize their capabilities.

## Author contributions

Zahra Saadatidizaji, Iman Zare, and Mingzhen Zhang: conceptualization, investigation, validation, writing – original draft, and writing – review & editing. Shahrzad Zirak Hassan Kiadeh, Mojdeh Mirshafiei, Amin Farahani, Zahra Sadat, and Hooman Aghamirza Moghim Aliabadi: investigation, validation, visualization, and writing – original draft. Mohammad Mahdavi: investigation, validation, and writing – original draft. Reza Eivazzadeh-Keihan and Amir Kashtiaray: conceptualization, validation, project administration, supervision, and writing – review & editing. Parisa Rezvani, Aisan Yahyapoor, Amirali Vaziri, and Hooriehsadat Amini: investigation, validation, and writing – original draft. Meng Yun: conceptualization, validation, supervision, and writing – review & editing.

## Conflicts of interest

The authors declare no competing interests.

## Abbreviations

ASCs: Adipose tissue-derived stem cells
AMC: Adriamycin
AL: Albumin
ARS: Alizarin Red S
ALP: Alkaline phosphatase
AAO: Anodic aluminum oxide
BC: Bacterial cellulose
BET: Brunauer–Emmett–Teller
β-TCP: β-Tricalcium phosphate
CFP: Capillary flow porometry
CFMEs: Carbon fiber microelectrodes
CNT: Carbon nanotubes
CHA: Catalytic hairpin assembly
Cat B: Cathepsin B
CFX: Cephalexin
CS: Chitosan
ChOx: Cholesterol oxidase
CIP: Ciprofloxacin
CD: Circular dichrogram
COL: Collagen
CG: Colloidal gold
COF: Covalent organic framework
CD: Cyclodextrin
dECM: Decellularized extracellular matrix
DXMT: Dexamethasone
DSC: Differential scanning calorimetry
DMOG: Dimethyloxalylglycine
DOX: Doxorubicin
DDSs: Drug delivery systems
ECL: Electrochemiluminescence
ePTFE: Expanded polytetrafluoroethylene
ECM: Extracellular matrix
FA: Folic acid
FDM: Fused deposition modeling
GelMA: Gelatin methacrylate
GIC: Glass ionomer cement
GCE: Glassy carbon electrode
AuNPs: Gold nanoparticles
NPAu: Gold nanoporous
GA: Graphene aerogel
GO: Graphene oxide
GOQDs: Graphene oxide quantum dots
GFP: Green fluorescent protein
GG: Gum ghatti
HE: Heparin-eluting
HFFC: Hemostatic film-forming composition
HPCs: Hollow polymeric capsules
hADSCs: Human adipose mesenchymal stem cells
HAEC: Human aortic endothelial cells
HDMVE: Human dermal microvascular endothelial
hPDLCs: Human periodontal ligament cells
HAS: Human serum albumin
HAP: Hydroxyapatite
HPMC: Hydroxypropyl methylcellulose
IBU: Ibuprofen
ISFs: Interstitial fluids
KTP: Ketoprofen
KEGG: Kyoto encyclopedia of genes and genomes
LDH: Lactate dehydrogenase
LOD: Limit of detection
LDM: Low-temperature deposition modeling
MNCs: Magnetic nanoclusters
MDD: Modified drug delivery
mMWCNTs: Modified multi-walled carbon nanotubes
MS: Mesoporous silica
MSC: Mesenchymal stem cell
MOFs: Metal–organic frameworks
MTX: Methotrexate
MB: Methylene blue
NPs: Nanoparticles
NPMs: Nanoporous materials
NPAM: Nanoporous alumina membranes
NAA: Nanoporous anodic alumina
NFRCSs: Nanoporous fiber-reinforced composite scaffolds
NPG: Nanoporous gold
nMN: Nanoporous microneedle
NS: Nanosheet
NAs: Neutralizing antibodies
OD: Optical density
OMCs: Ordered mesoporous carbons
ONC: Ordered nanoporous carbon
OCN: Osteocalcin
pAP: *p*-Acetamidophenol
P-MCSs: Phosphorus-doped microporous carbon spheroidal structures
PANI: Polyaniline
PAEK: Polyaryletherketone
PCL: Polycaprolactone
PCMs: Polycarbonate membranes
PEDOT: Poly(3,4)ethylene dioxythiophene
PEG: Polyethylene glycol
PEO: Polyethylene oxide
PLA: Polylactic acid
PTFE: Polytetrafluoroethylene
PtNPs: Platinum nanoparticles
PU: Polyurethane
PUU: Polyurethane urea
PVF: Polyvinyl formal
QUC: Quercetin
rMSCs: Rat mesenchymal stem cells
rGO: Reduced graphene oxide
RhB: Rhodamine B
SEM: Scanning electron microscopy
SCs: Schwann cells
SPE: Screen-printed electrode
SNM: Silica nanoporous membrane
SF: Silk fibroin
AgNPs: Silver nanoparticles
NaCMC: Sodium carboxymethylcellulose
SPS: Spark plasma sintering
SPA: *Staphylococcus aureus* protein A
scCO_2_: Supercritical carbon dioxide
3D: Three-dimensional
TEAGs: Tissue-engineered arterial grafts
TMP: Trimethoprim
TR: Trypsin
WCA: Water contact angle
XRD: X-ray diffraction

## Data Availability

No primary research results, software or code have been included and no new data were generated or analysed as part of this review.
